# Fiber-Optic Pressure Sensors: Recent Advances in Sensing Mechanisms, Fabrication Technologies, and Multidisciplinary Applications

**DOI:** 10.3390/s25206336

**Published:** 2025-10-14

**Authors:** Yihang Wang, Botong Chen, Guirong Wu, Chenyang Xue, Libo Gao

**Affiliations:** 1 Pen-Tung Sah Institute of Micro-Nano Science and Technology, Xiamen University, Xiamen 361102, China; wangyihang2022@163.com (Y.W.); chenbotong2025@163.com (B.C.); wuguirong@stu.xmu.edu.cn (G.W.); 2 Shenzhen Research Institute of Xiamen University, Xiamen University, Shenzhen 518000, China; 3 School of Aerospace Engineering, Xiamen University, Xiamen 361102, China

**Keywords:** fiber-optic pressure sensors, distributed detection, multimodal integrated sensors, wearable devices

## Abstract

Fiber-optic sensing (FOS) technology has emerged as a cutting-edge research focus in the sensor field due to its miniaturized structure, high sensitivity, and remarkable electromagnetic interference immunity. Compared with conventional sensing technologies, FOS demonstrates superior capabilities in distributed detection and multi-parameter multiplexing, thereby accelerating its applications across biomedical, industrial, and aerospace fields. This paper conducts a systematic analysis of the sensing mechanisms in fiber-optic pressure sensors, with a particular focus on the performance optimization effects of fiber structures and materials, while elucidating their application characteristics in different sensing scenarios. This review further examines current manufacturing technologies for fiber-optic pressure sensors, covering key processes including fiber processing and packaging. Regarding practical applications, the multifunctional characteristics of fiber-optic pressure sensors are thoroughly investigated in various fields, including biomedical monitoring, industrial and energy monitoring, and wearable devices, as well as aerospace monitoring. Furthermore, current challenges are discussed regarding performance degradation in extreme environments and multi-parameter cross-sensitivity issues, while future research directions are proposed, encompassing the integration and exploration of novel structures and materials. By synthesizing recent advancements and development trends, this review serves as a critical reference bridging the gap between research and practical applications, accelerating the advancement of fiber-optic pressure sensors.

## 1. Introduction

As a key medium for sensing the physical world, the core function of a pressure sensor is to accurately convert the pressure signal applied to it into a quantifiable and transmittable electrical or optical signal. In many fields, such as modern industrial automation, aerospace, biomedical diagnosis, environmental monitoring, and energy exploration, the real-time and accurate acquisition of pressure parameters plays an irreplaceable fundamental role in system safety, process optimization, and control. For example, real-time pressure monitoring is the key to ensuring flight safety within an aeroengine [1]; accurate intravascular blood pressure data is the core basis for guiding clinical treatment in medical monitoring [2]; and downhole pressure distribution directly determines recovery efficiency and operation safety in oil and gas exploitation [3]. Therefore, pressure-sensing technology with high performance and strong reliability is developing rapidly. Its accuracy, stability, environmental adaptability, and integration are being continuously improved. This progress plays a key role in driving scientific advancement and upgrading industries. It also brings important scientific value, along with significant economic and social benefits.

According to its core principle, traditional pressure sensors mainly include piezoresistive, piezoelectric, capacitive, and resonant types [4,5,6,7]. Although piezoresistive sensors are mature in technology and low in cost, their outputs are easily affected by temperature drift, and there are significant safety hazards in strong electromagnetic interference (EMI) or flammable and explosive environments. Piezoelectric sensors have a fast dynamic response, but they cannot detect static or quasi-static pressure and have a high output impedance and complex signal conditioning circuit. Capacitive sensors have high sensitivity and low power consumption characteristics, but they are sensitive to environmental humidity and dust pollution. The packaging process is demanding, and the long-term stability is easily restricted by the aging of dielectric materials. Resonant sensors have high precision, but their structure is delicate and fragile, their manufacturing cost is high, and their anti-vibration and impact ability is generally insufficient. These inherent defects severely limit the potential of traditional sensors for applications in extreme environments, implantable medical monitoring, and other cutting-edge fields.

Fiber-optic pressure sensors [8] are a new generation of sensing technology developed to break through the above bottlenecks. Its main advantage is the use of light waves as information carriers. It employs quartz glass or specially designed polymer optical fibers for transmission and sensing. This technology is inherently safe and highly resistant to electromagnetic interference. It can also withstand high temperatures, high pressure, and harsh chemical environments. In addition, it offers excellent electrical insulation and enables long-distance, distributed measurements. FOPS can be divided into two types—fiber grating type [9] and interferometric type [10]—according to their sensing mechanism. A fiber grating sensor works by detecting changes in the grating period or the effective refractive index of the fiber core caused by external pressure. These changes result in a shift of the reflected or transmitted wavelength. By analyzing the wavelength shift, pressure can be accurately measured. This type of sensor offers advantages such as wavelength-based encoding and easy multiplexing for networked systems. Interferometric sensors operate based on the principle of optical interference. When pressure is applied, it alters either the cavity length or the refractive index of the fiber. This leads to changes in the intensity or phase of the interference light. By detecting this change, pressure information is retrieved, usually with extremely high sensitivity. Other types of sensors based on fiber bending losses [11,12], unique optical properties of microstructure fibers [13] and photonic crystal fibers [14] are also included. These diverse sensing mechanisms provide a wealth of technological options for different application scenarios.

In order to comprehensively and systematically discuss the research progress in the field of optical fiber pressure sensors, this review will follow a clear logical framework. First, we designed a table based on the analysis of sensing requirements. Key performance specifications for fiber-optic pressure sensors, such as pressure range, sensitivity, resolution, and response time, are summarized along with other critical parameters that define sensor applicability and performance (Table 1). This facilitates the comparison of different fiber-optic pressure sensor designs. In Section 2, the fundamental physical sensing mechanism of the fiber-optic pressure sensor is thoroughly investigated, focusing on fiber grating and interferometric sensors, which are widely used. This lays a theoretical foundation for understanding the following contents. Section 3 turns to the materials [15,16,17] that constitute the sensor, discusses the key materials used to manufacture the sensor unit in detail, and analyzes its performance characteristics and application scenarios. Section 4 focuses on the key to realizing the sensor’s function, systematically combs through the mainstream fabrication technology and technology of the fiber-optic pressure sensor, covering the specific methods of grating writing [18,19] and microcavity construction [20,21]. Section 5 expands the field of vision to practical applications and demonstrates the unique value and successful practice of optical fiber pressure sensors in aerospace [22], biomedical [23,24], industrial control [25], geological structure monitoring [26], and intelligent structure health diagnosis [27]. Finally, Section 6 will provide an objective analysis of the main challenges currently faced by optical fiber pressure sensors. These include improving sensitivity and resolution, minimizing temperature-stress cross-sensitivity, achieving miniaturization and large-scale production, reducing manufacturing costs, and developing efficient and reliable demodulation systems. In addition, we will explore future development directions. Key trends include the use of novel sensing materials and micro/nano structural designs, the integration of multiple sensing functions, and the development of flexible and wearable devices, as well as the application of AI for signal processing and fault diagnosis. It will also discuss potential strategies for large-scale integration and application of these technologies. This review holds important academic and practical value. From a scholarly perspective, it systematically addresses the entire technical chain of optical fiber pressure sensors, covering fundamental physical principles, material selection, fabrication techniques, and application development. This comprehensive overview provides researchers with a clear understanding of the field’s evolution and helps to identify key research hotspots and challenges. In terms of technology, analyzing and comparing different sensing methods and manufacturing processes can inspire new sensor designs that perform well and cost less. This is especially useful for developing sensors for harsh environments, biocompatible devices, and small-scale integration. It also encourages collaboration between fields like optics, materials science, microfabrication, and biomedical engineering. In terms of applications, this review thoroughly summarizes the real-world performance and challenges of sensors across various scenarios. It offers technical evaluations and practical guidance to assist industry in selecting and optimizing sensing systems, as well as in developing emerging markets. This helps to speed up the transition from laboratory research to large-scale industrial use. Ultimately, the review aims to serve as a key reference for researchers and engineers, supporting the ongoing advancement of optical fiber pressure-sensing technology toward greater accuracy, enhanced robustness, broader applicability, and increased intelligence. It provides a strong foundation for both scientific progress and societal development.

## 2. Sensing Mechanism of Optical Fiber Pressure Sensors

The core function of an optical fiber pressure sensor is to convert external mechanical pressure into measurable changes in the optical signals transmitted through the fiber. This process relies on the fiber’s unique waveguide structure and the interaction between light and matter. In fiber-optic pressure sensors, external pressure is typically converted into mechanical deformation through structures such as diaphragms, capillaries, or cavities, which then act on the optical fiber to achieve optical signal modulation. For FBG sensors, most studies employ structures such as diaphragms, levers, or elastic membranes to convert external pressure into axial strain on the optical fiber, thereby enhancing the sensor’s sensitivity. Xu et al. pointed out that the pressure response of a bare FBG is extremely low (approximately 3.04 pm/MPa) and emphasized the importance of mechanical amplification structures [28]. For example, the coaxial steel tube structure proposed by Nellen et al. and the metal diaphragm structure developed by Qi Jiang et al. enhanced the pressure sensitivity to levels above 20 pm/MPa [29,30]. Her and Weng employed an epoxy diaphragm with an embedded FBG, where varying diaphragm thicknesses resulted in sensitivities ranging from 43.7 to 175.5 pm/kPa, indicating that diaphragm thickness is a critical parameter [31]. In contrast, interferometric sensors rely on the deflection of a diaphragm under pressure, which induces changes in the cavity length and subsequently alters the position or phase of interference fringes. These structural designs directly determine the sensor’s sensitivity, linearity, and operational range.

**Figure 1:**
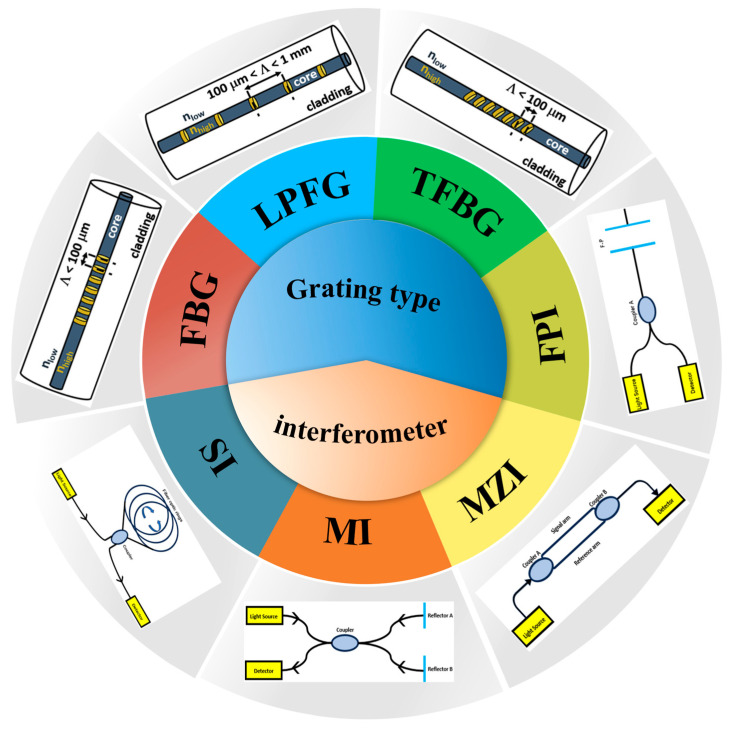
Mechanism of optical fiber pressure sensors.

A typical optical fiber consists of a central core, surrounded by cladding with a lower refractive index, and an outer protective coating. There are two main types of sensing mechanisms: grating and interferometric (Figure 1). Grating-type sensing is realized by creating precise periodic variations in the refractive index within the fiber core. These structures act as wavelength selectors, causing strong reflection or mode coupling at specific wavelengths based on Bragg conditions. When pressure is applied, axial strain and transverse deformation alter the grating period and effective refractive index, leading to a measurable shift in the characteristic wavelength. Pressure information can be demodulated by detecting the wavelength modulation amount. Interferometric sensing relies on the interference of light. When pressure is applied to the sensing region, it causes physical deformation and changes the fiber’s refractive index, which modulates the phase of the sensing light. This phase-modulated light interferes with a reference beam, resulting in shifts of interference fringes or changes in the interference spectrum (such as drift or intensity variations). By precisely measuring these changes, the applied pressure can be determined with high sensitivity. Whether based on wavelength-selective grating technology or phase-interference of optical waves, the core principle is the same: converting pressure-induced mechanical deformation into measurable changes in specific optical parameters within the fiber. These changes are then demodulated using optical techniques to achieve accurate pressure sensing. Together, these two mechanisms form the fundamental technical basis of optical fiber pressure sensing.

### 2.1. Fiber Bragg Grating Pressure Sensor

**Figure 2:**
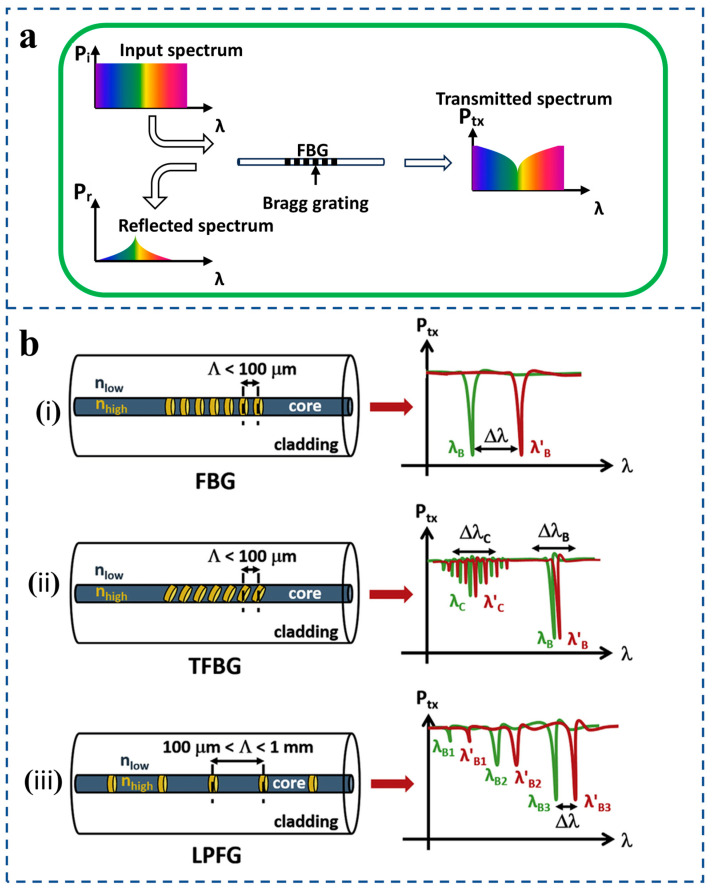
(**a**) Spectrum diagram of the fiber Bragg grating sensing principle. (**b**) (**i**) Schematic diagram of FBG structure and its spectral response (reprinted with permission from [33] © Elsevier, 2019). (**ii**) Schematic diagram of TFBG structure and its spectral response (reprinted with permission from [33] © Elsevier, 2019). (**iii**) Schematic diagram of LPFG structure and its spectral response (reprinted with permission from [33] © Elsevier, 2019).

The fiber Bragg grating type pressure sensor is a sensor that uses the grating principle to detect pressure changes (Figure 2a). Fiber grating is a structure formed by periodically changing the refractive index in the optical fiber. For a fiber grating pressure sensor, its periodic structure can reflect light of a specific wavelength. When the external pressure acts on the fiber, it will cause the grating period and refractive index of the fiber to change, thus causing a shift of the reflected wavelength. By measuring the wavelength change of the reflected light, information on pressure changes can be obtained. There are many kinds of fiber-optic sensors. The origin of fiber grating can be traced back to the 1970s [32], and the commonly used grating pressure sensors in practical applications are fiber Bragg grating (FBG), long-period fiber grating (LPFG), tilted fiber Bragg grating (TFBG), and other fiber grating pressure sensors. A schematic diagram of a fiber Bragg grating-based structure and its corresponding spectral response are presented (Figure 2b) [33]. The central operational parameter of the FBG, λB, is defined as the wavelength at which phase-matched coupling between counter-propagating core modes occurs, resulting in strong reflection. It is given by the formula λ_B_ = 2n_eff_·Λ. When it becomes λ′_B_ due to external influences, its offset Δλ_B_ can be used to measure parameters such as strain and temperature. For TFBG and LPFG, the coupling mechanism produces a series of cladding mode resonance wavelengths λ_rm_ (or λ_C_), which are sensitive to the variation in the external refractive index, and the offset Δλ_C_ (i.e., changing from λ_C_ to λ′_C_) is an important sensing index. λ_B1_, λ_B2_, and λ_B3_ are used for distinguishing multiple gratings written into the same fiber to realize multi-point distributed sensing.

#### 2.1.1. FBG Pressure Sensor

FBG operates by creating a periodic variation in the refractive index within the core of an optical fiber. This structure acts as a selective mirror, reflecting a very specific wavelength of light known as the Bragg wavelength. The precise value of this wavelength is determined by the period of the grating and the effective refractive index of the core, making it highly sensitive to changes in physical conditions. Consequently, any external strain that stretches the grating or temperature shift that alters the material’s index will cause a measurable shift in the reflected wavelength, which is the fundamental principle behind its use as a sensor for mechanical and thermal monitoring.

FBG pressure sensors have compact size, immunity to electromagnetic interference, excellent safety, distributed sensing, and many other inherent advantages. Xu et al. [34] studied two types of FBG pressure sensors and proposed that FBG pressure sensors will have broad prospects for development in the future. Qiao et al. [35] experimentally achieved high-temperature and -pressure measurements up to 400 °C and 100 MPa by combining FBG sensors with suitable metal sensors. Xue et al. [36] proposed a fiber Bragg grating temperature and pressure sensor based on a carbon fiber tube, which can work stably in 0~150 °C and 0~80 MPa environments, and the maximum pressure sensitivity can reach −50.02 pm/MPa while showing a good linear response. It meets the precision requirement in the process of downhole exploitation and provides an experimental basis for the design of optical fiber sensors for high temperature and high pressure in oil and gas downholes.

#### 2.1.2. LPFG Pressure Sensor

LPFG functions through a periodic refractive index modulation with a much longer spatial period. Unlike FBGs, this longer period enables co-directional coupling, where light from the fundamental core mode is transferred forward into specific co-propagating cladding modes. These cladding modes are then attenuated as they travel through the fiber, resulting in one or more broad attenuation bands in the transmission spectrum. The resonant wavelengths of these bands are highly sensitive to bends in the fiber, changes in temperature, and, most prominently, the refractive index of the immediate external environment.

The essential difference lies in the grating period: FBG employs short-period gratings (typically ~0.5 μm) to satisfy Bragg reflection, while LFBG uses long-period gratings (hundreds of micrometers) to couple core modes to cladding modes. In 1996, A. M. Vengsarkar et al. [37] successfully fabricated the first hydrogen-loaded LPFG by using ultraviolet light through an amplitude mask. Two years later, Davis et al. [38,39] proposed for the first time in 1998 that focused CO_2_ laser pulses could be used to write directly on optical fibers, and laser-fabricated LPFG devices were realized. In 2003, Rao et al. [40] developed a new type of LPFG optical fiber sensor based on high-frequency CO_2_ laser pulse writing. The fiber can be directly processed using the laser’s monochromaticity and high power output, which greatly promotes the preparation and accuracy of LPFG. In 2015, Tang et al. [41] proposed and realized a gas pressure sensor, laser-induced long-period fiber grating in hollow core photonic bandgap fiber, which can be used to develop a promising gas pressure sensor with a sensitivity of −137 pm/MPa. In 2016, Y. Tsutsumi et al. [42] proposed and demonstrated a simple optical gas pressure sensor with a long-period fiber grating (LPFG) fabricated from heat-shrink tubing. In 2024, C.J. H et al. [43] proposed LPFG based on microporous PDMS filling. Experimental results showed that after PDMS filling, LPFG had improved its sensitivity to gas pressure to −2.23 nm/MPa.

#### 2.1.3. TFBG Pressure Sensor

TFBG is a variant where the grating planes are intentionally angled relative to the fiber’s axis. This tilt breaks the symmetry found in standard FBGs and fundamentally changes the light interaction within the fiber. While some light is still coupled backward into the core mode, a significant portion is radiated outward and couples into forward-propagating cladding modes. This creates a complex transmission spectrum featuring numerous narrow resonance bands. The key operational principle is that the properties of these cladding modes are extremely sensitive to the refractive index of the material surrounding the outside of the fiber.

TFBG is a variant of the original FBG concept and, by tilting the grating plane relative to a perpendicular line to the fiber axis, periodic perturbations interact with core-guided light to obtain characteristics distinct from ordinary gratings.

The TFBG model first appeared in 1990, when G. Meltz et al. [44] proposed that TFBG can enhance and control radiation mode coupling. Since then, a large number of scholars have carried out targeted research on TFBG, and TFBG sensing technology has ushered in rapid development. In 2007, E. Chehur et al. [45] proposed an optical fiber sensor capable of distinguishing temperature and strain using a single fiber Bragg grating. This technique takes advantage of the core–cladding mode coupling of TFBG. Monitoring core–core coupling resonance and core–cladding mode coupling resonance of TFBG spectra can separate temperature- and strain-induced wavelength shifts.

In 2011, Shao et al. [46] proposed a novel lateral force sensor based on a core-offset tilted fiber Bragg grating (TFBG), in which the lateral force is determined by the differential reflected power between the cladding mode and Bragg mode. The sensor responds monotonically with lateral force increasing from 0 N to 1.75 N. The sensitivity of this TFBG sensor can be customized by selecting different values of core offset. The simple differential power detection method enables cost-effective implementation of sensor systems and is immune to environmental and system fluctuations. The TFBG pressure sensor uses the principle of fiber Bragg grating to convert pressure signals into measurable optical signals, which has unique advantages, especially high accuracy, long-distance transmission, and environmental adaptability.

### 2.2. Optical Fiber Interferometric Pressure Sensor

Interferometric fiber-optic pressure sensor converts pressure signals into optical phase changes by using the optical interference effect and then realizes high-sensitivity measurement. Common interferometric fiber-optic pressure sensors include fiber-optic Fabry–Pérot interferometer (FPI) pressure sensors, fiber-optic Mach–Zehnder interferometer (MZI) pressure sensors, fiber-optic Michelson interferometer (MI) pressure sensors, and fiber-optic Sagnac interferometer (SI) pressure sensors. Fiber-optic interferometers have received considerable attention for their potential sensing applications in refractive index, temperature, pressure, and strain measurements. Compared with grating pressure sensors, interferometric fiber-optic pressure sensors have more extensive applications due to their higher sensitivity, more flexible measurement methods, and wider adaptability.

#### 2.2.1. Fabry–Pérot Interferometer (FPI) Pressure Sensors

The fiber-optic Fabry–Pérot sensor is a sensing mechanism based on multi-beam interference. The origin of its technical development can be traced back to the invention of the multi-beam interferometer at the end of the 18th century. Early research focused on bulky Fabry–Pérot interferometers. Until the 1980s, with the progress of micro–nano processing technology [47,48], the optical Fabry–Pérot interferometer achieved a breakthrough in miniaturization. This technological innovation has promoted the wide application of Fabry–Pérot sensors in the field of sensing and detection and has spawned a variety of miniaturized Fabry–Pérot sensors with different structures.

**Figure 3:**
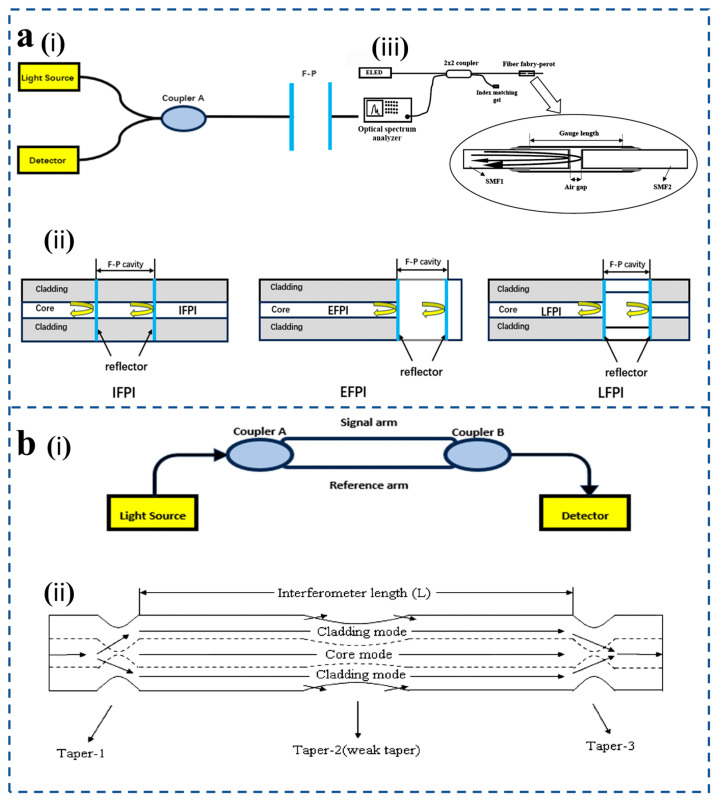
(**a**) (**i**) Fabry–Pérot interferometer basic schematic diagram. (**ii**) Classification of FPI pressure sensors: IFPI, EFPI, LFPI. (**iii**) Principle of EFPI sensor (reprinted with permission from [53] © Elsevier, 2006). (**b**) (**i**) Mach–Zehnder interferometer basic schematic diagram. (**ii**) Schematic diagram of an online Mach–Zehnder modulator based on three tapers and a transmission spectrum with interaction length L of ~60 mm (reprinted with permission from [55] © Optical Society of America, 2011).

The Fabry–Pérot (F-P) interferometer is a typical multi-beam interferometer [49]. Its basic principle is the same as that of a multi-beam interferometer of parallel plates [Figure 3a(i)]. After the light transmitted by the light source is reflected and transmitted by two parallel and highly reflective mirrors in the resonant cavity many times, the change in the cavity length will cause the phase difference of the light source after transmission in the mirror, so the transmitted light will interfere. According to different fiber Fabry–Pérot cavities, fiber Fabry–Pérot sensors can be divided into the following three types [Figure 3a(ii)]: intrinsic Fabry–Pérot interferometer (IFPI) [50], extrinsic Fabry–Pérot interferometer (EFPI) [51], and linear Fabry–Pérot interferometer (LFPI) [52] The main difference lies in the construction of the Fabry–Pérot cavity: the cavity of an IFPI is formed by the intrinsic structure of the optical fiber itself, the cavity of an EFPI consists of an air gap between the fiber end face and an external reflective surface, while an LFPI is constructed by fusion-splicing a segment of hollow-core fiber between two single-mode fibers to form a fixed linear air cavity.

In recent years, fiber-optic FPI has attracted much attention due to its wide range of applications. For example, Aref et al. [53] presented an extrinsic fiber Fabry–Perot interferometer (EFPI) sensor for pressure measurement with low sensitivity variation. An EFPI sensor operates by detecting the interference pattern between two light beams reflected from a tiny air gap, which changes when the gap is altered [Figure 3a(iii)].

Currently, the high corrosion resistance and high temperature resistance of optical fiber F-P pressure sensor make it an ideal pressure measurement tool in high-temperature and high-pressure environments. S.M.N. Rao [54] proposed a new technique for creating pressure sensors based on optical F-P cavities at the tip of optical fibers, overcoming the size limitation of traditional pressure sensors. The sensor forms an interference structure by processing micro cavities inside the optical fiber and belongs to the IFPI type. This progress demonstrates that fiber-optic Fabry–Pérot pressure sensors (including EFPI and IFPI types) will have broader application prospects. With the continuous development of new materials and new technologies, the performance of optical fiber F-P pressure sensors will be further improved, especially in sensitivity, accuracy, response speed, and so on.

#### 2.2.2. Mach–Zehnder Interferometer (MZI) Pressure Sensors

The Mach–Zehnder interferometer (MZI) fiber-optic pressure sensor is a kind of pressure sensor based on the MZI principle. A conventional Mach–Zehnder interferometer is illustrated, in which the light source is split into two beams after passing through the first coupler [Figure 3b(i)]. One fiber serves as a reference arm, and the other as a sensing arm. When external pressure acts on the sensing arm, it will change the phase of light in the sensing arm. When the light of the sensing arm and the light of the reference arm pass through the second coupler, interference will occur due to the phase difference of the two beams of light. The change in interference fringes can be detected by a photodetector, and the pressure parameters we want to detect can be obtained by analyzing the interference spectrum signal with a photodetector.

In order to improve the sensitivity of the MZI fiber-optic pressure sensor, Wu et al. [55] introduced a Mach–Zehnder interferometer based on three cascaded SMF tapers. A Mach–Zehnder interferometer is formed with taper-1 and taper-3 as splitters and combiners, and a weak taper in the middle is used to increase the evanescent field of the cladding mode excited by taper-1 in the external medium [Figure 3b(ii)]. Increasing the interaction between the interferometer and the external medium ultimately improves sensitivity.

MZI fiber-optic sensors require splitters and combiners to split the input optical signal into two distinct optical paths (core and cladding) and then recombine them. Typical techniques for making splitters/combiners include misaligned splice joints [56], peanut structures [57], and laser irradiation [58]. In terms of pressure sensors, microcavity MZI pressure sensors can easily obtain higher sensitivity [59,60]. However, special tools, such as femtosecond lasers are needed to fabricate microcavities, which increases the research and development cost of sensors. For the pressure sensor developed by core fusion or taper, the development method is relatively simple, but it needs to add auxiliary materials to achieve pressure sensing [61].

#### 2.2.3. Michelson Interferometer (MI) Pressure Sensors

**Figure 4:**
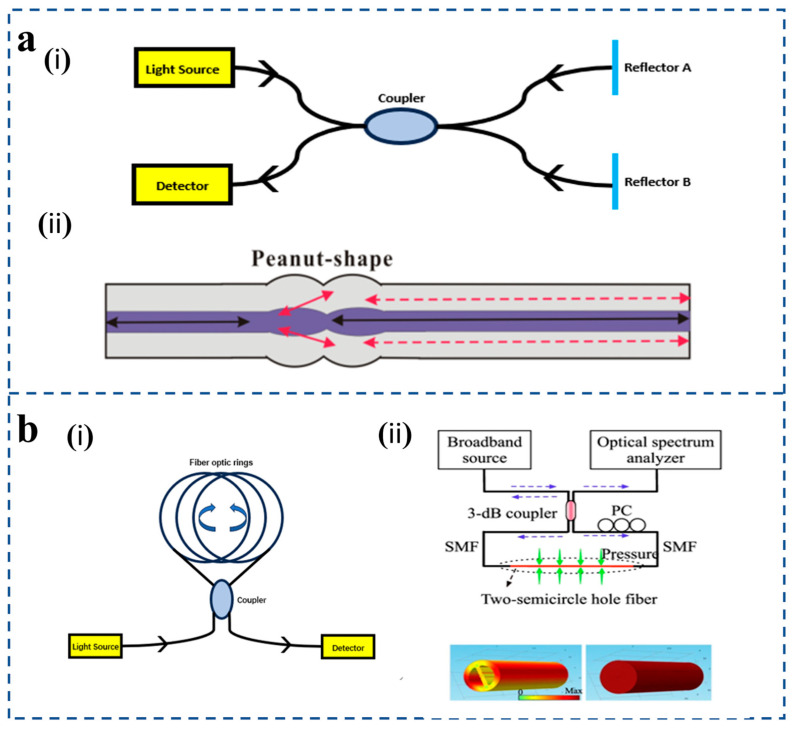
(**a**) (**i**) Michelson interferometer schematic diagram. (**ii**) The peanut-shape-based MI schematic (reprinted with permission from [57] © MDPI). (**b**) (**i**) Sagnac interferometer schematic diagram. (**ii**) Schematic of the Signac interferometer setup, and the SI transmission spectrum using 1.058 m of the TSH-MOF (reprinted with permission from [64] © Springer Nature, 2018).

The Michelson interferometric fiber-optic pressure sensor is a pressure-sensing device designed based on the Michelson interferometer principle [Figure 4a(i)]. The light emitted by the laser enters the input fiber, and the light source passes through the coupler and is divided into two beams of light, which are sent, respectively, into two single-mode fibers with basically the same length, and the end faces of the two single-mode fibers are coated with high reflectivity modes. One beam of light passes through the sensing arm and is reflected by a movable mirror at the end, usually used to detect changes in the external environment; the other part of the light passes through the reference arm and is reflected by a fixed mirror at the end, which remains unchanged in a constant environment. The two reflected beams are combined by the coupler to cause interference, and the intensity change of the interference signal is measured by the detector [62]. For the problem of connecting the sensor to the single-mode optical fiber, specifically, the light from the broadband light source is transmitted to the sensor through the single-mode optical fiber. After modulation in the Fabry–Pérot cavity, the reflected light carrying the external parameter information returns along the original path and is finally received and analyzed by the demodulation system. This all-fiber optical connection method not only ensures the matching of optical field modes and low transmission loss, but also avoids additional alignment steps, significantly improving the stability and reliability of the sensor.

Both fiber MI and fiber MZI are dual-beam interferometers. And in terms of fiber components, interference principles, measurement parameters, etc., they are also very similar. However, MZI uses the transmission spectrum, while MI uses the reflection spectrum. At the same time, since MI uses reflection mode, it is compact and convenient in practical use and installation. The multiplexing capability of multiple sensors in parallel is another advantage of MI. However, the fiber length difference between the reference and sensing arms of MI must be adjusted within the coherence length of the source [63]. Wu et al. [57] have shown that a new peanut fiber structure can excite higher-order cladding modes and recouple cladding modes to core modes, so inline MI in SMF can also act as a splitter and combiner by using only one peanut structure [Figure 4a(ii)].

#### 2.2.4. Sagnac Interferometer (SI) Pressure Sensors

The Sagnac interferometric (SI) fiber-optic pressure sensor is a pressure-sensing device designed based on the Sagnac interferometer principle [Figure 4b(i)]. SI is widely used in various sensing environments because of its high sensitivity, easy preparation, and exceptional performance in sensing acoustic or dynamic pressure fields through their response to the rate of change in pressure. Incident light emitted by the light source passes through the optical fiber coupler and is divided into two beams of light, which are transmitted clockwise and counterclockwise, respectively. When the sensing element is affected by pressure, the two beams of light will be affected by each other [65]. Because the optical path is different, a phase difference will occur between the clockwise and counterclockwise transmitted lights. When the two beams of light meet again in the coupler, interference signals will be sent to the photodetector. The magnitude of the measured physical quantity is reflected by monitoring the phase change of the interference signal.

Fiber-optic Sagnac interferometers have been used in pressure sensors and other sensor applications due to their unique advantages of simple design, ease of manufacture, and low sensitivity to environmental acquisition noise. Z. Liu et al. [64] propose a sensitive large dynamic range pressure sensor based on a novel birefringent microstructured optical fiber (MOF) deployed in a Sagnac interferometer configuration [Figure 4b(ii)]. It should be noted that all interferometric sensors inherently exhibit a limited dynamic range because their output phase is periodic with 2π, which limits the measurement of dynamic pressure.

### 2.3. Optical Fiber Vernier Effect Pressure Sensor

**Figure 5:**
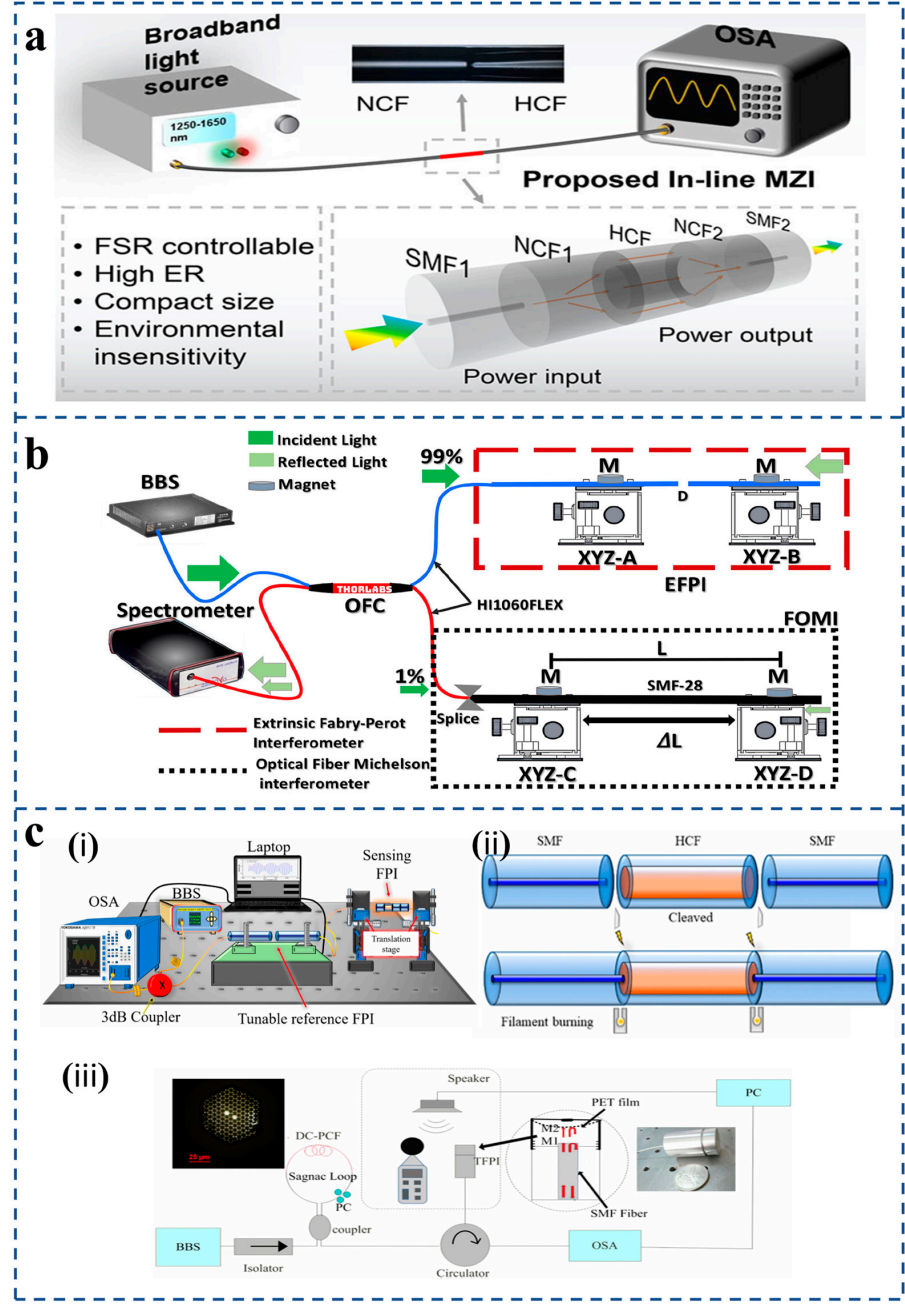
(**a**) Schematic diagram of the NCF-HCF-NCF in-line MZI with experimental setup; the proposed MZI is connected to a broadband light source and spectral analyzer (reprinted with permission from [66] © IEEE). (**b**) Strain gauge device using a fiber-optic Michelson interferometer (sensing element) and an exogenous Fabry–Pérot interferometer (reprinted with permission from [67] © Taylor & Francis Group, 2022). (**c**) (**i**) Splicing and cleaving setup for fabrication of the proposed sensor (reprinted with permission from [69] © MDPI). (**ii**) The fabrication process of sensing FPI cleaved fibers and spliced fibers (reprinted with permission from [69] © MDPI). (**iii**) Schematic diagram of experimental device and the simulation result of the vernier effect (reprinted with permission from [70] © IEEE).

In recent years, the vernier effect has been introduced into optical fiber interferometric sensors in order to improve sensitivity. The vernier effect is a signal amplification technique where the interference of two cascaded resonators with slightly different spectral responses produces an envelope signal whose shift is greatly magnified compared to that of a single resonator. M. Dai et al. [66] proposed a compact in-line Mach–Zehnder interferometer (MZI) by sandwiching a hollow capillary fiber (HCF) between two lengths of coreless fiber (NCF) as a reference arm in a sensing system based on the vernier effect (Figure 5a). The NCF-HCF-NCF structure has the advantages of small volume, good spectral characteristics, and being insensitive to the surrounding environment.

As a simple and effective method to improve sensitivity, the vernier effect has been widely studied and applied in optical fiber sensors in recent years (Figure 5b). The strain-sensing device shown employs both a fiber-optic Michelson interferometer (sensing element) and an exogenous Fabry–Pérot interferometer, both of which excite the vernier effect [67]. This design improves sensor performance. In addition, the pressure sensor proposed by Xu et al. [68] consists of two cascade Fabry–Pérot interferometers separated by a long section of single-mode fiber to create a vernier effect. The pressure sensor has the advantages of high sensitivity, compact structure, and simple manufacture and has potential application prospects in the fields of biomedicine and marine exploration. F. Mumtaz et al. [69] proposed a high-sensitivity strain sensor based on a tunable cascaded Fabry–Pérot interferometer and demonstrated it experimentally [Figure 5c(i)]. A broadband source with a narrow bandwidth of 1530 nm to 1610 nm, optical spectrum analyzer, a laptop for the acquisition of data, and a 3 dB coupler are used to obtain the reflection spectrum of the proposed sensor: a highly sensitive strain sensor based on tunable cascaded Fabry–Perot interferometers (FPIs). The fabrication process is as follows: The sensing FPI is formed by splicing of SMF–HCF–SMF in the form of concatenation. The SMF is cleaved and then spliced with a piece of HCF [Figure 5c(ii)]. Then, the HCF is cleaved at 400 μm with the LDC-401 cleaver, which has the ability to cleave at a micro-scale with high precision. After cleaving the HCF, it is spliced with SMF so that the SMF–HCF–SMF structure is formed.

S. Wang et al. [70] proposed a fiber-optic acoustic sensor with real-time controllable sensitivity based on the optical vernier effect [Figure 5c(iii)]. At the same time, high sensitivity and a large sound pressure measurement range are obtained, breaking the contradiction between sensitivity and measurement range. The sensor has the advantages of real-time controllable sensitivity, high sensitivity, and large dynamic range, which is further beneficial to its practical application.

## 3. Materials of Fiber Pressure Sensors

The performance limits and application scope of sensors are largely determined by the properties and structural innovations of their core materials. Recent advancements in material systems are pushing beyond the physical constraints of traditional silica fibers by employing multifunctional composites and microstructured designs to simultaneously enhance pressure sensitivity, environmental resilience, and signal transmission efficiency. In fiber grating sensors, material research focuses on enhancing the responsiveness of the photosensitive layer and ensuring the stability of the grating region. For Fabry–Pérot cavity sensors, advancements target the selection of cavity media and optimization of reflective interfaces to balance optical precision and mechanical reliability. The need for sensors to work in extreme environments is driving continuous improvements in materials, such as heat-resistant bases, radiation-resistant coatings, and smart materials that can respond to changes. By combining material design, function, and structure, sensors can now perform reliably under tough conditions like deep-sea high pressure or sudden temperature changes in aerospace. At the same time, these advances are opening up new possibilities for using sensors in life science applications. This section mainly introduces the core materials used in fiber-optic pressure sensors. It should be noted that the fiber material used in this study is not a standard single-mode fiber (SMF), but a specially designed fiber for high-sensitivity pressure-sensing requirements. These types of optical fibers usually have unique microstructures, which efficiently convert external pressure changes into optical signal changes by enhancing the interaction between light and mechanical deformation, thereby achieving high sensitivity and precision measurement of small pressures.

### 3.1. Fiber Bragg Grating Pressure Sensor Materials

In the design of fiber Bragg grating (FBG)-based pressure sensors, the material system functions as a multifunctional and synergistic ensemble. The optical base material—silica—forms the optical fiber body that transmits light signals, serving as the operational foundation of the sensor. To achieve high-sensitivity detection, a flexible matrix—often polymer—is engineered into deformable structures (such as thin films or cavity fillers) whose role is to directly transduce external pressure and efficiently transfer it to the internal FBG [71]. Leal. A.G. et al. [72] experimentally demonstrated the potential of PDMS for higher pressure sensitivity, which was confirmed in pressure characterization tests (showing a pressure sensitivity of 1.4 nm/kPa). To further enhance performance, functional sensing materials such as graphene are incorporated to improve specific sensitivities (e.g., enabling self-temperature compensation) or introduce new capabilities such as chemical sensing [73]. Finally, the entire sensing unit is encapsulated within protective materials—such as glass sealants [74] or packaging polymers [75]—that form a robust housing essential for isolating the sensor from complex external environments, thereby ensuring stable and reliable operation. Through the precise collaboration of these materials, the system accurately converts physical pressure into shifts in optical wavelength signals.

In the fiber grating type pressure sensor, a refractive index difference is formed between the cladding and the fiber core, so that the optical signal is confined to the fiber core through reflection. The Bragg wavelength of FBG is determined by the effective refractive index of the core and grating period, while the effective refractive index is directly affected by the refractive index of the cladding. Cladding plays a key role in fiber grating pressure sensors, directly affecting the sensitivity, stability, environmental adaptability, and pressure transmission mechanism of the sensor, so it is very important to select suitable cladding materials.

The fiber material that constitutes the fiber grating is generally composed of an isotropic core and cladding. Introducing anisotropic materials into the fiber structure has a significant impact on the transmission characteristics, dispersion characteristics, and power distribution between the core and cladding of the fiber. The magnitude of the impact depends on the refractive index distribution of the fiber material.

The classification of cladding materials includes not only isotropic materials and anisotropic crystalline materials, but also a variety of other material types, each suitable for different sensing scenarios based on its physicochemical properties. Among anisotropic crystal materials, uniaxial crystal materials [76] are widely used because of their unique advantages. For example, the strong piezoelectric effect and high strain optical coefficient in the uniaxial crystal material LiTaO_3_ can detect small strain changes with high sensitivity through measurable birefringence changes and phase modulation [77]. The combination of high strain response rate and stable crystal structure makes it an ideal material for high-sensitivity strain sensing in harsh environments. The uniaxial crystal materials used for fiber core or cladding mainly include NaNO_3_, LiTaO_3_, LiNbO_3_, potassium dihydrogen phosphate [78], etc. (Table 2). In 2004, Zhang Xiaoping et al. [79] theoretically studied the electro-optic effect and elastic-optic effect in chirped fiber gratings with uniaxial cladding and obtained the curves of Bragg wavelength and reflectivity versus electric field intensity and strain field when LiNb03, LiTa03, and KDP were used as cladding, respectively. M.A.I. Jahan et al. [80] proposed a new method to improve the sensitivity of pressure sensors based on fiber Bragg gratings. The material of choice is a cyclic olefin copolymer called TOPAS with a core refractive index of 1.53 and a cladding of 1.525. Strain or temperature changes the Bragg wavelength by compressing the grating and changing the effective index, and it was observed that TOPAS FBG responds more effectively in sensing applied strain than silica FBG, indicating higher sensitivity. In addition, the coating material of fiber grating is important to the performance, reliability, and application environment adaptability of fiber grating. The main function of the coating layer is to protect the fiber grating from mechanical damage, chemical corrosion, and environmental factors. Coating materials usually have polymer coatings, metal coatings, etc. For different applications and requirements in different fields, it is necessary to select suitable coating materials for optical fibers.

**Table 2:** FBG pressure sensors’ cladding materials.

Cladding Material	Refractive Index	Wavelength	Reference
NaNO_3_	1.58–1.336	632.8 nm	[78]
LiTaO_3_	2.176–2.180	633 nm	[78]
LiNbO_3_	2.29–2.20	633 nm	[78]
Potassium dihydrogen phosphate (KDP)	1.51–1.47	633 nm	[78]
TOPAS cyclic olefin copolymer	1.525	1550 nm	[80]

**Figure 6:**
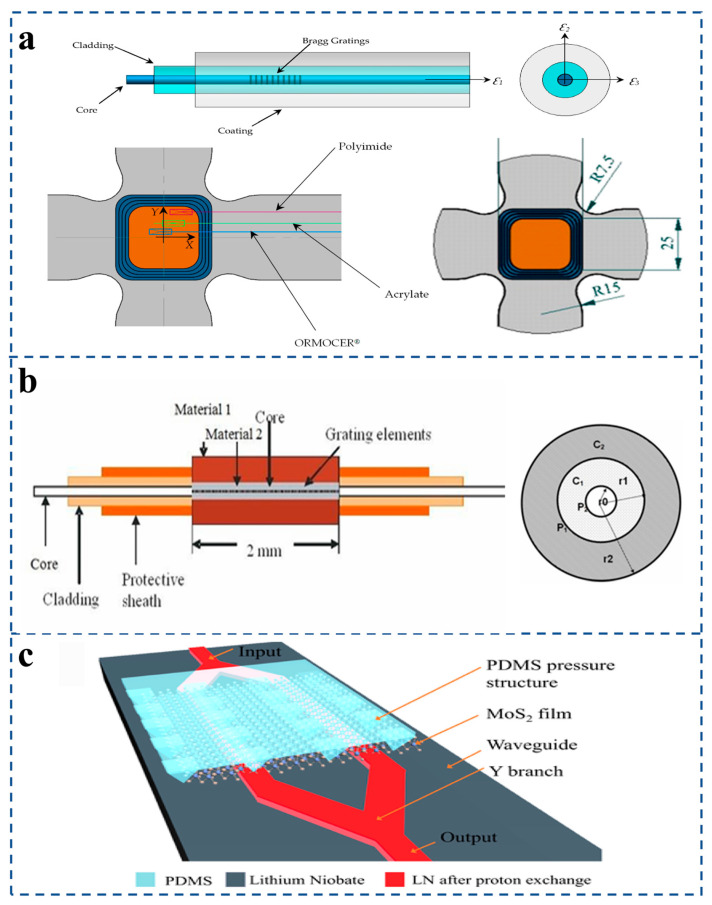
(**a**) FBG sensors installed on the specimen central zone. Specimen dimensions and detailed views. Parts of the fiber Bragg gratings sensors (FBGSs) (reprinted with permission from [82] © MDPI). (**b**) Dual-layer metal-coated FBG sensor (DMCFBG). Model DMCFBG. Stress variations for primary coating and secondary layer thickness (reprinted with permission from [83] © IOP). (**c**) Three-dimensional schematic diagram of MoS_2_ optical pressure sensor (reprinted with permission from [85] © Optica Publishing Group, 2022).

FBG sensors used for structural health monitoring will be affected by the simultaneous presence of multi-directional strain [81]. M. González-Gallego et al. [82] developed an experimental method involving a biaxial test scheme for cruciform samples, three of which were embedded FBG sensors coated with polyimide, acrylate, and ORMOCER polymers (Figure 6a). Experimental verification shows that the strain sensitivity factor depends on the sensor coating material. Ramalingam et al. [83] proposed that the thickness of the primary and secondary coatings of the double-layer metal-coated FBG sensor is 125 μm and 625 μm, respectively (Figure 6b).

The results show that lead-and-indium-coated DMCFBG sensors exhibit higher sensitivity below 20 K, while indium-coated DMCFBG sensors exhibit higher sensitivity in the lower temperature range of 50 K–4.2 K. Furthermore, G. Kaur et al. [84] proposed a cladding-etched fiber Bragg grating sensor to monitor chemicals for use in civil structures. In addition, indium tin oxide is coated on the clad etched FBG sensor to improve sensitivity. It has been observed that sensors coated with indium tin oxide are very sensitive in terms of chemical detection and induced wavelength shifts of Ca(OH)_2_ at 12 nm and 10 nm. For other classes of coatings, Li et al. [85] proposes a sensitivity-enhanced optical pressure sensor based on molybdenum disulfide (MoS_2_). The sensing mechanism converts pressure into a measurable wavelength shift in an optical spectrum. Compared to a graphene-based film, the MoS_2_ film demonstrates a superior light modulation effect (Figure 6c). Experimental results show the sensor achieves a high sensitivity of 96.02 nm/kPa within a 0–0.6 kPa range, significantly outperforming typical optical pressure sensors. This design shows high potential for ultra-sensitive pressure detection applications.

### 3.2. Optical Fiber Interferometric Pressure Sensor Materials

Interferometric-based fiber-optic sensors are widely used for pressure measurement in various applications due to their compact size, high sensitivity, immunity to electromagnetic interference, and survivability in harsh environments. Various materials, such as silica [86,87,88,89], polymers [90,91,92,93], metals [94,95], graphene [96], and silk fibroin, have been used to fabricate elastic diaphragms for use as sensing elements. The performance of various membrane-based fiber-optic pressure sensors discussed in this section is summarized, highlighting key metrics such as pressure range, sensitivity, and resolution (Table 3).

**Table 3:** Interferometric fiber pressure sensor materials and properties.

Material	Primary Function	Sensitivity	Test Range	Reference
Silica	Transmitting optical signals	9.48 pm/kPa	0~200 kPa	[86]
		11 nm/kPa	0~100 kPa	[87]
		12.4 nm/kPa	6.9~48.3 kPa	[88]
		3.4 nm/kPa	0~1 MPa	[89]
Polymer	Detecting and transmitting pressure signals	100 pm/kPa	100~175 kPa	[90]
		52.143 nm/Mpa	0.1~0.7 Mpa	[91]
		20.63 nm/MPa	0~2 MPa	[92]
		395 pm/kPa	0~30 kPa	[93]
Graphene	Enhance specific sensitivity	39.4 nm/kPa	0~50 kPa	[94]
Metal	Construct an interference cavity	1.6 nm/kPa	0~50 psi	[95]
		19.5 nm/kPa	0~100 kPa	[96]

The material system of fiber-optic interferometric pressure sensors (e.g., Fabry–Pérot (F-P) type) constitutes a complex multi-material microsystem centered around a miniature optical interference cavity. This stands in sharp contrast to fiber Bragg grating (FBG) sensors, which primarily rely on external structures to introduce axial strain. The design of such sensors not only involves metal or polymer diaphragms as deformable interfaces but also incorporates a wide range of functional materials: high-strength coatings such as polyimide are used to enhance the mechanical and environmental durability of the optical fiber [97]; silicone oil or optical gel serves as cavity-filling material to ensure uniform and isotropic pressure transmission while protecting internal structures; high-reflectivity interfaces are formed inside the cavity through magnetron-sputtered gold/silver thin films or deposited silica dielectric mirrors—a core process not found in FBG-based sensors. Furthermore, fillers such as silica nanoparticles or graphene are incorporated into polymer matrices (e.g., PDMS) to actively tailor mechanical properties like Poisson’s ratio and Young’s modulus, thereby optimizing linearity and suppressing hysteresis in the sensing characteristics. Therefore, the material structure core of interferometric sensors is a precise collaborative system of “membrane cavity mirror functional composite material”, which has much higher material diversity, interface complexity, and functional integration than FBG, aiming to achieve direct and high-precision tuning of the optical cavity length parameter.

**Figure 7:**
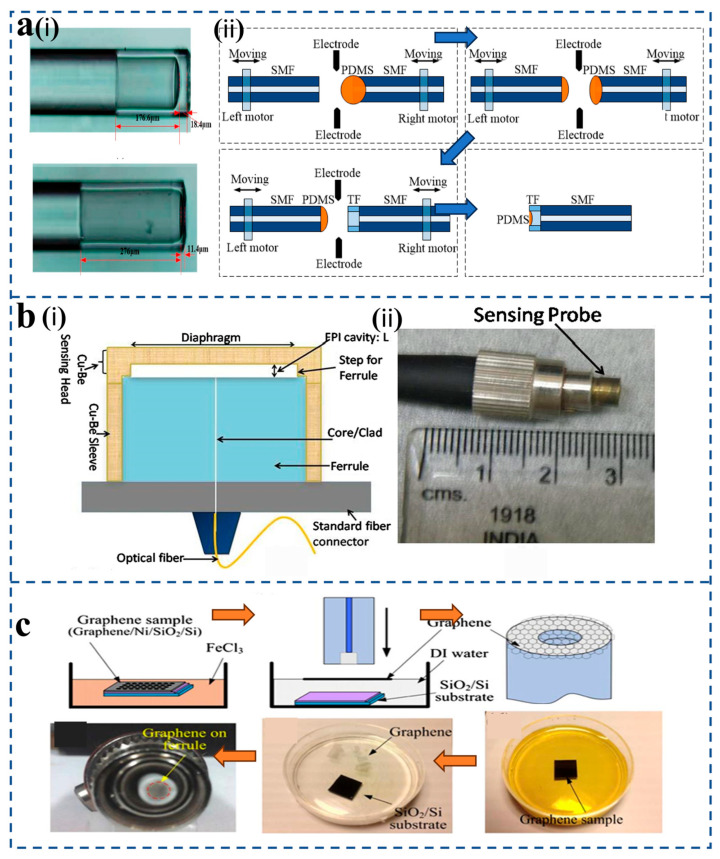
(**a**) (**i**) Fabricated fiber Fabry–Pérot sensors (reprinted with permission from [90] © MDPI). (**ii**) Fabrication process of the PDMS immersed fiber sensor (reprinted with permission from [90] © MDPI). (**b**) (**i**) Schematic diagram of the EFPI acoustic sensor based on gold film (reprinted with permission from [95] © Elsevier, 2020). (**ii**) Sensor appearance and dimensions (reprinted with permission from [95] © Elsevier, 2020). (**c**) Fabrication process of the fiber-tip microcavity with a graphene diaphragm (reprinted with permission from [96] © Optical Society of America, 2012).

Luo et al. [90] fabricated a thin-film immersion F-P fiber-optic pressure sensor using PDMS material [Figure 7a(i)]. By analyzing the relationship between the PDMS diffusion rate and viscosity, the PDMS was cured in an oven to a specific degree to accurately control its diffusion rate and diffusion length within the optical fiber tube [Figure 7a(ii)]. The volume of PDMS in the fiber tube was controlled by various transfer methods to form the minimum PDMS film thickness. A new type of optical fiber Fabry–Pérot pressure sensor is formed, which is sensitive to external pressure parameters. The optimized film thickness can be reduced to 20 μm, and the optical fiber pressure sensor has a sensitivity up to 100 pm/kPa. PDMS thin film has many advantages, such as low cost, good controllability, etc., and has good application value in high-sensitivity pressure and acoustic detection.

Ghildiyal et al. [95] presents a novel Fabry–Pérot pressure sensor [Figure 7b(i)] with a corrosion-resistant copper–beryllium alloy diaphragm, fabricated via Diamond Turning Machining to achieve high precision, and reports a sensitivity of over 1 µm/bar. This study introduces a novel three-dimensional monolithic sensing head that integrates the pressure-sensitive diaphragm and the Fabry–Pérot cavity step, which is welded to a sleeve to precisely align the optical fiber and form the interferometer [Figure 7b(ii)].

Ma et al. [96] innovatively combined graphene with optical fiber to design a high-sensitivity graphene diaphragm fiber-optic tip pressure sensor (Figure 7c). This study uses graphene thin film to construct a miniature optical fiber pressure sensor. Graphene acts as a light reflector, forming a high-precision Fabry–Pérot interferometer. The pressure sensitivity of graphene-based sensors exceeds 39.4 nm/kPa.

## 4. Manufacturing Technology of Optical Fiber Pressure Sensors

The way optical fiber pressure sensors are made is shifting significantly, from traditional approaches toward more precise and integrated techniques. This shift plays a key role in how accurate the sensors are and how well they work in different environments. Modern manufacturing methods bring together knowledge from several advanced fields, including optical engineering, materials science, and micro- and nanofabrication. At the heart of these sensors are two main types of sensing structures: fiber gratings and Fabry–Pérot cavities. Each comes with its own technical challenges. For fiber gratings, the focus is on how accurately the grating is written and how well it is packaged. In contrast, Fabry–Pérot cavities require more careful control over the size of the cavity and the quality of the reflective surfaces. Ultrafast laser processing makes it possible to build tiny optical structures with great accuracy. At the same time, using new functional materials helps sensors to stay stable even in tough or extreme environments. Thanks to progress in micro- and nanomanufacturing, sensors are becoming smaller and more compact, which opens up new uses in things like consumer electronics and healthcare devices. As the manufacturing process becomes more standardized and automated, fiber-optic pressure sensors are starting to move out of the lab and into real industrial production. To make that happen, we not only need to solve technical problems in the process, but also set up clear standards and reliable testing systems.

### 4.1. Manufacturing Method of Fiber Grating Pressure Sensor

In order to realize the efficient fabrication of fiber grating pressure sensors, researchers have explored and proposed a variety of advanced manufacturing technologies. These technologies can be divided into two broad categories, non-laser manufacturing and laser manufacturing [98], depending on how they work. Laser fabrication techniques, such as CO_2_ laser writing [99] or femtosecond laser writing [100], and non-laser fabrication techniques such as oxyhydrogen flame melting [101], mechanical microbending [102], acousto-optic modulation [103], and arc discharge [104]. At present, laser manufacturing technology is the most mainstream manufacturing method of fiber grating pressure sensors.

**Figure 8:**
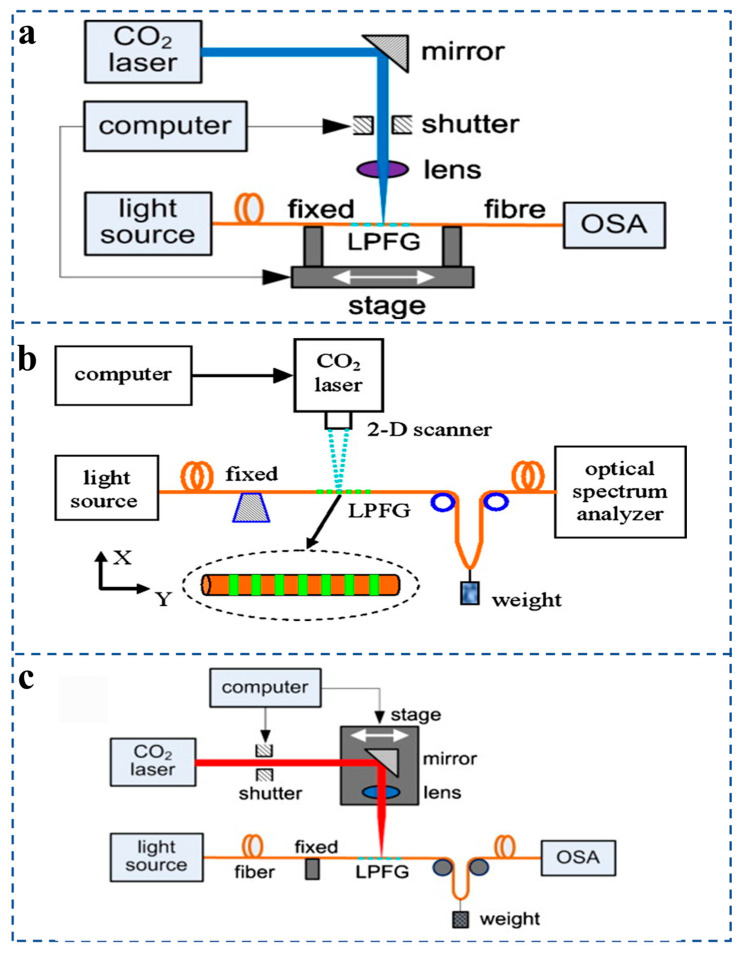
(**a**) Schematic diagram of a normal LPFG fabrication system based on the typical point-to-point technique employing a CO_2_ laser (reprinted with permission from [105] © AIP Publishing). (**b**) Schematic diagram of LPFG fabrication system based on two-dimensional scanning of focused high-frequency CO_2_ laser pulses (reprinted with permission from [105] © AIP Publishing). (**c**) Schematic diagram of an improved LPFG fabrication system employing a CO_2_ laser (reprinted with permission from [105] © AIP Publishing).

For example, Wang outlines three primary CO_2_ laser irradiation techniques for fabricating long-period fiber gratings (LPFGs). The first technique (Figure 8a) uses a typical point-to-point method where the fiber is periodically moved along its axis while a computer-controlled shutter allows the laser to irradiate it intermittently, though this can cause vibration issues. The second approach (Figure 8b) employs two-dimensional scanning of high-frequency CO_2_ laser pulses across a stationary fiber under tension, eliminating the need for synchronized movement and reducing vibration, thereby producing high-quality gratings with low insertion loss. The third, improved system (Figure 8c) combines elements of both, using a linear motor stage to shift a focused laser beam via a mirror and cylindrical lens without moving the fiber, thus enhancing stability and repeatability while allowing continuous grating writing during fiber drawing.

Furthermore, J. Guo et al. [106] demonstrated a femtosecond (fs) laser-written cladding waveguide phase-shifting structure for high-accuracy blood pressure (BP) measurements. This structure consists primarily of phase-shifted FBG written into the cladding waveguide of a single-mode fiber (SMF) encapsulated in flexible polydimethylsiloxane (PDMS). Experimental results show that the proposed sensor shows a high degree of consistency with commercial electronic sphygmomanometers and achieves considerable accuracy, with an average difference of only 1.357 ± 1.74 mmHg.

Through the innovation of the sensor preparation process, optical fiber pressure sensors can achieve high sensitivity, strong anti-electromagnetic interference ability, good anti-corrosion performance, and so on.

### 4.2. Manufacturing Method of Optical Fiber Interference Pressure Sensor

**Figure 9:**
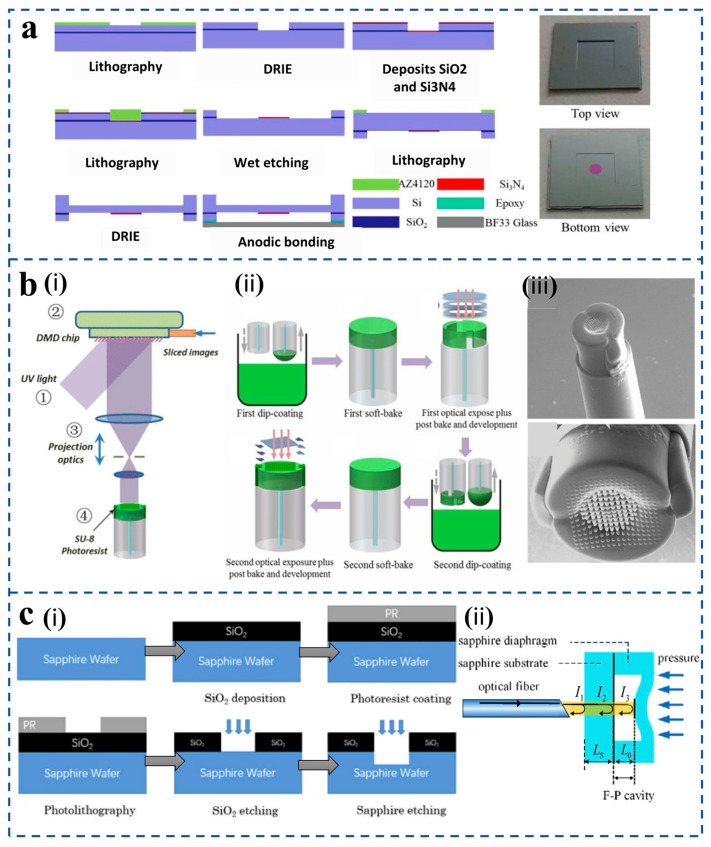
(**a**) The fabrication process and photos of the fabricated pressure sensor (reprinted with permission from [88] © MDPI). (**b**) (**i**) Schematic of the optical in situ μ-printing technology (reprinted with permission from [107] © IEEE). (**ii**) Optical printing flow for the fiber-tip FPI pressure sensor (reprinted with permission from [107] © IEEE). (**iii**) Scanning electron images of the fiber-tip FPI pressure sensors with light scatter (reprinted with permission from [107] © IEEE. (**c**) (**i**) Fabrication flow chart of the sapphire sensitive diaphragm (reprinted with permission from [108] © Optical Society of America, 2010). (**ii**) Schematic of the sapphire fiber-optic pressure sensor (reprinted with permission from [108] © Optical Society of America, 2010).

Qi et al. [88] presents a compact optical MEMS pressure sensor based on Fabry–Pérot (FP) interference, designed for wind pressure monitoring on transmission line towers due to its immunity to electromagnetic interference (Figure 9a). The sensor integrates a MEMS sensing chip (comprising an FP cavity and a square silicon diaphragm fabricated from a Silicon-on-Insulator (SOI) wafer), a vertical-cavity surface-emitting laser (VCSEL), and a photodiode (PD) into a miniature housing. The fabrication process involves lithography and deep reactive ion etching (DRIE) to define the FP cavity and diaphragm structure, deposition of SiO_2_ and Si_3_N_5_ layers to achieve a reflectivity of ~0.03, and final anodic bonding with a glass wafer to form a sealed FP cavity with a 25 μm gap. Experimental results show the sensor has a measurement range of 0–700 Pa, a sensitivity of 115 nA/kPa, and an accuracy of 3.12% FS, making it suitable for low-pressure wind monitoring applications.

As a new and growing technology, 3D printing offers several advantages when it comes to making optical fibers. It allows for quick prototyping of complex microstructures, can precisely produce tiny parts like fiber gratings and microfluidic channels, and helps to simplify the steps involved in traditional manufacturing methods. Wu et al. [107] developed an air cavity fiber Fabry–Pérot interferometric pressure sensor based on 3D-printing technology. An in-house-developed optical 3D μ-printing system was employed to fabricate the optical fiber-tip FPI pressure sensors [Figure 9b(i)]. The fabrication process follows a two-step exposure strategy: the first step involves printing the suspended SU-8 diaphragm, and the second step simultaneously seals the air cavity and forms the light-scattering structure [Figure 9b(ii)]. The light-scattering and sealing components are precisely positioned, demonstrating the high alignment accuracy of the optical printing system [Figure 9b(iii)]. By printing such a microstructure on the end face of the optical fiber, the sensitivity is effectively improved, and the influence of the surrounding environment on the sensor is reduced. The Fabry–Pérot interferometer has a linear response to pressure change in the range of 0~700 kPa, and its sensitivity is 2.93 nm/MPa.

Chemical etching is also one of the core manufacturing technologies for fiber-optic sensors. Shao et al. [108] proposed an external Fabry–Pérot interferometer (EFPI) pressure sensor based on an optimized wet etching process using all-sapphire substrates, aiming to improve the quality of the interference signals. The preparation process of the proposed wet anisotropic etching is illustrated [Figure 9c(i)]. A commercially obtained C-plane (0001) sapphire wafer (210 µm thick, RMS roughness ~0.33 nm) was used as the substrate. A 20 µm thick SiO_2_ film was deposited via plasma-enhanced chemical vapor deposition. A 2 mm × 2 mm square was then patterned using photolithography and transferred into the SiO_2_ layer by reactive ion etching to create a hard mask. After etching the sapphire, the SiO_2_ mask was removed with HF solution, yielding a patterned sapphire diaphragm [Figure 9c(ii)].

## 5. Application of Optical Fiber Pressure Sensors

**Figure 10:**
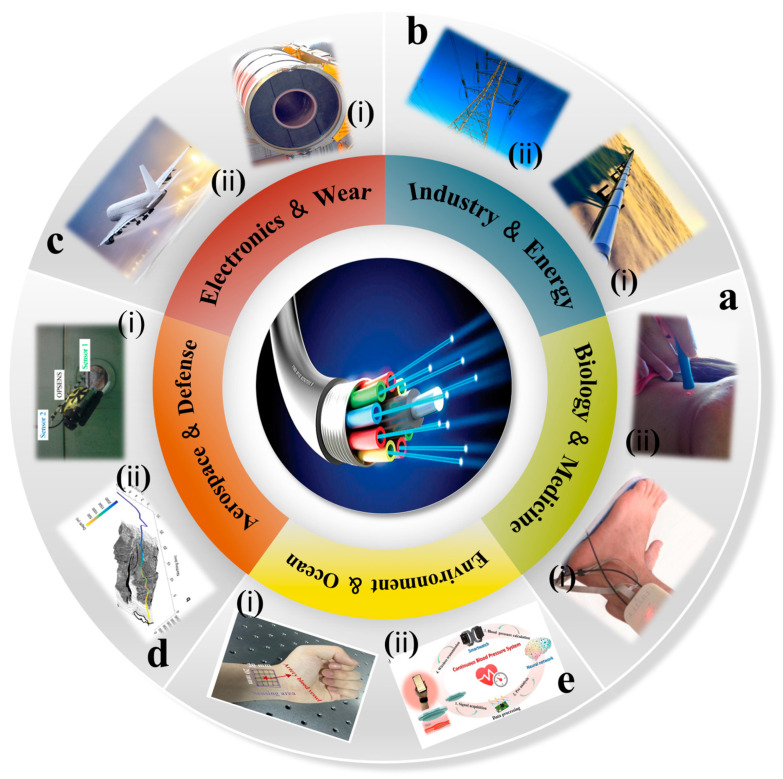
Various field scenarios of optical fiber pressure sensors. (**a**) Biomedical field. (**i**) Foot pressure detection (reprinted with permission from [109] © Elsevier, 2019). (**ii**) Carotid artery pressure detection (reprinted with permission from [110] © Elsevier, 2016). (**b**) Industrial and energy field. (**i**) Application of Fiber Optic Pressure Sensors in Pipelines. (**ii**) Application of Fiber Optic Pressure Sensors in Power Towers. (**c**) Aerospace and defense field. (**i**) Application of Fiber Optic Pressure Sensors in defense. (**ii**) Application of Fiber Optic Pressure Sensors in aerospace. (**d**) Environment and ocean field. (**i**) Underwater depth monitoring (reprinted with permission from [111] © MDPI). (**ii**) Seismic surveillance (reprinted with permission from [112] © Springer Nature). (**e**) Consumer electronics and smart wearables field (reprinted with permission from [113] © Springer Nature). (**i**) The monitoring system consists of an optical fiber sensor, signal acquisition, data processing, blood pressure calculation, smartwatch terminal, and mobile phone. (**ii**) The sensing area with sixteen grids on the wrist.

Fiber-optic pressure sensors have become key sensing technologies in multidisciplinary applications due to their unique physical characteristics and customizable design (Figure 10). In the biomedical field, their biocompatibility and millimeter-level miniaturization capability enable precision medical applications such as intravascular pressure monitoring and intracranial pressure dynamic tracking, while avoiding the electromagnetic interference risk of traditional electronic sensors. In industrial settings, the all-fiber design allows sensors to operate reliably under tough conditions like high pressure and strong corrosion. This makes them ideal for tasks such as monitoring stress in petrochemical pipelines or measuring internal pressure in turbine engines, helping to make industrial equipment smarter. For environmental monitoring, distributed optical fiber sensing networks can gather data on deep-sea pressure changes and seismic waves at the same time by using underwater optical cables. This provides valuable tools for studying marine climate and giving early warnings for geological disasters. In aerospace, these sensors take advantage of being lightweight and resistant to radiation. They have been successfully used in harsh conditions, like tracking aerodynamic loads on aircraft surfaces and checking the seal integrity inside spacecraft cabins. Innovative uses in consumer electronics stand out as well. For example, flexible fiber arrays combined with smart textiles have been used to create smart sports shoes that monitor pressure distribution on the soles in real time, with sampling rates of 1 kHz or higher. This work highlights the special benefits and new contributions that optical fiber pressure sensors bring to all these areas.

### 5.1. Biomedical Field

As people pay more attention to their health today, real-time monitoring of physiological signals has become very important for understanding individual health status. These signals not only serve as key references for medical diagnosis and treatment but also act as early signs for proactive health management. Because physiological signals are often subtle and continuous, sensors need to be highly sensitive and consistent. Fiber-optic pressure sensors are well suited for this role since they resist electromagnetic interference and can comfortably attach to the body, ensuring accurate and reliable data. They are used in areas like monitoring breathing, heartbeats, blood pressure, and walking patterns, which help with early disease detection and support recovery after surgery.

**Figure 11:**
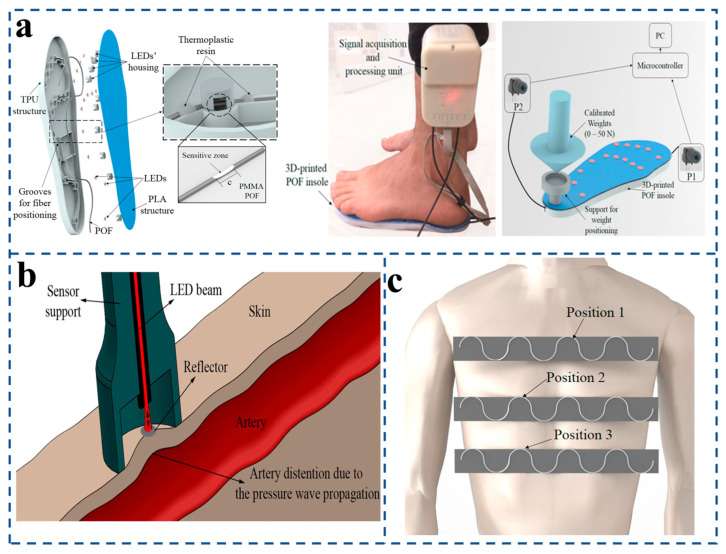
(**a**) Foot pressure optical fiber sensor (reprinted with permission from [109] © Elsevier, 2019). (**b**) Carotid artery dilation waves measuring fiber-optic sensor (reprinted with permission from [110] © Elsevier, 2016). (**c**) An intelligent optical pressure sensor that simultaneously monitors breathing and heart rate (reprinted with permission from [115] © Elsevier, 2018).

Arnaldo G. et al. [109] developed a polymer fiber-optic pressure sensor for static and dynamic evaluation of plantar pressure and ground reaction forces. They propose fifteen intensity-based sensors multiplexed into a single polymer fiber (Figure 11a). It can accurately analyze the weight of the human body and the pressure change of the sole during walking. At the same time, the high accuracy of the experiment was verified by drawing a pressure map based on the perception of the subject’s plantar pressure. C. Leitão et al. [110] designed a low-cost fiber-optic sensor using a polymer fiber-optic photodiode and a polylactic acid sheath to make a light probe for measuring carotid artery dilation waves (Figure 11b). Xue et al. [114] developed a human respiration monitoring system based on a wearable tilt fiber grating sensor that provides a repeatable, highly sensitive, and realistic time response to curvature changes during respiration. The breathing changes of the human body under different motion conditions can be accurately detected. Important physiological parameters such as respiratory rate, exhalation, and inhalation duration obtained from the system software provide valuable data to assist in the clinical diagnosis of respiratory diseases. The sensor response curve is plotted by capturing the frequencies of three different breathing patterns (rapid breathing, normal breathing, and deep breathing) by the TFBG sensor. Different breathing patterns can be successfully identified from the sensor response curve (i.e., the output of the sensor’s light amplitude and frequency). These preclinical results support future clinical validation studies in larger and broader cohorts. In 2018, Arnaldo et al. [115] proposed a smart textile embedded solution that can simultaneously monitor breathing and heart rate (Figure 11c). Breathing rate (BR) and heart rate (HR) can be obtained without the human body being affected by body motion and at different positions on the user’s chest; the results show that even if the user is performing periodic body motion, the errors of HR and BR are less than 4 heartbeats per minute and 2 breaths per minute, respectively. It can be easily applied to remotely monitor patients at home without interfering with their daily activities.

Overall, these studies highlight the ability of fiber-optic pressure sensors to detect physiological signals, improve high sensitivity, robustness, and stability for accurate medical detection, and lay the foundation for more medical fiber-optic sensor research.

### 5.2. Industrial and Energy Field

Fiber-optic pressure sensors are becoming extremely useful tools in industries like energy and manufacturing. Because they do not rely on metal or electricity to work, they are not affected by electromagnetic interference, and they are safe to use in places where there is a risk of explosions or where the environment is tough, like in high temperatures or corrosive conditions. This makes them a great alternative to traditional electronic sensors, which can easily fail in these kinds of situations. These sensors can also be connected over long distances or built directly into equipment, making it possible to keep track of pressure in real time, for example, in energy pipelines, factory machines, or high-tech manufacturing setups. This helps to improve both safety and automation. Overall, these kinds of applications show how fiber sensors are not only reliable in extreme environments but also play a big role in pushing forward smarter and more advanced industrial systems.

**Figure 12:**
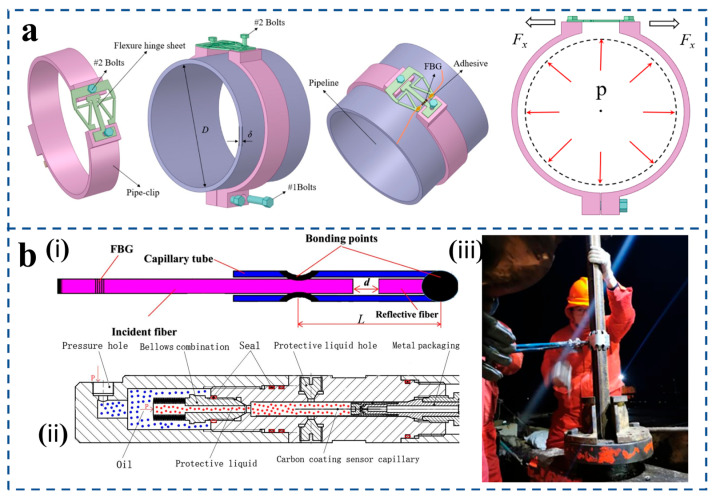
(**a**) Pipeline pressure detection FBG-sensitive structure (reprinted with permission from [116] © MDPI). (**b**) (**i**) Structure of the F-P cavity sensor with FBG cascaded (reprinted with permission from [117] © Elsevier, 2021). (**ii**) Schematic of the sensor encapsulation of using the bellows combination (reprinted with permission from [117] © Elsevier, 2021). (**iii**) Engineering application in Zhuangxi well (reprinted with permission from [117] © Elsevier, 2021).

Liu et al. [116] designed an FBG-sensitive structure based on a flexible hinge and applied it to pipeline pressure detection (Figure 12a). The team designed a structure based on flexible hinges and mechanical levers and mounted the fiber on top of the structure. When the pressure inside the tube rises, the wall expands, causing the structure to expand significantly and lengthen the fiber, resulting in a shift in the center wavelength of the fiber. The experimental results show that the strain sensitivity of the structure is 9.59 pm/µε, which is 11.51 times the surface strain of the pipe. Wang et al. [117] presents a carbon-coated and bellow-packaged optical fiber sensor for real-time high-pressure and high-temperature monitoring in downhole oil wells, utilizing an extrinsic Fabry–Pérot (F-P) interferometer for pressure sensing and a fiber Bragg grating (FBG) for temperature measurement and compensation. Key innovations include a carbon coating applied via CO_2_ laser-assisted CVD on the quartz capillary to prevent hydrogen diffusion and corrosion, and a hermetically sealed bellow–diaphragm package [Figure 12b(ii)] made of Inconel 625 to isolate the sensor from corrosive fluids while transferring external pressure. The sensor structure [Figure 12b(i)] features an F-P cavity formed within a capillary tube with a cascaded FBG. Laboratory tests demonstrated exceptional performance with >0.99999 linearity, 0.0396% F.S. repeatability, and 2.9 psi resolution. A successful field application in the Zhuangxi oil well [Figure 12b(iii)] confirmed its reliability for long-term downhole monitoring, accurately capturing dynamic pressure oscillations during oil production.

Optical fiber pressure sensors are playing an increasingly important role in modern industry and energy systems, thanks to their strong ability to adapt to harsh environments and provide stable, accurate monitoring. By overcoming the limitations of traditional sensors under extreme conditions, they offer a unique and valuable solution for improving industrial safety.

### 5.3. Environment and Ocean Field

In response to global climate change and the growing demand for marine resource exploration, optical fiber pressure sensors are emerging as a practical tool for monitoring extreme environments. They can withstand full ocean depth, resist salt spray corrosion, and remain stable over long periods. These features make them ideal for harsh marine conditions. Their passive, all-dielectric structure avoids electromagnetic interference, which helps to protect fragile ocean ecosystems. Unlike traditional electrical sensors, they do not introduce electromagnetic pollution. With the ability to support long-distance distributed sensing and detect multiple physical fields at once, these sensors enable the creation of intelligent monitoring networks. These networks can cover areas from the intertidal zone to deep-sea hydrothermal vents, and even polar ice caps. This advancement provides reliable data for understanding ocean dynamics. It also supports real-time geological hazard warnings and blue carbon ecosystem assessments. Altogether, it helps to improve our knowledge of Earth’s complex systems.

**Figure 13:**
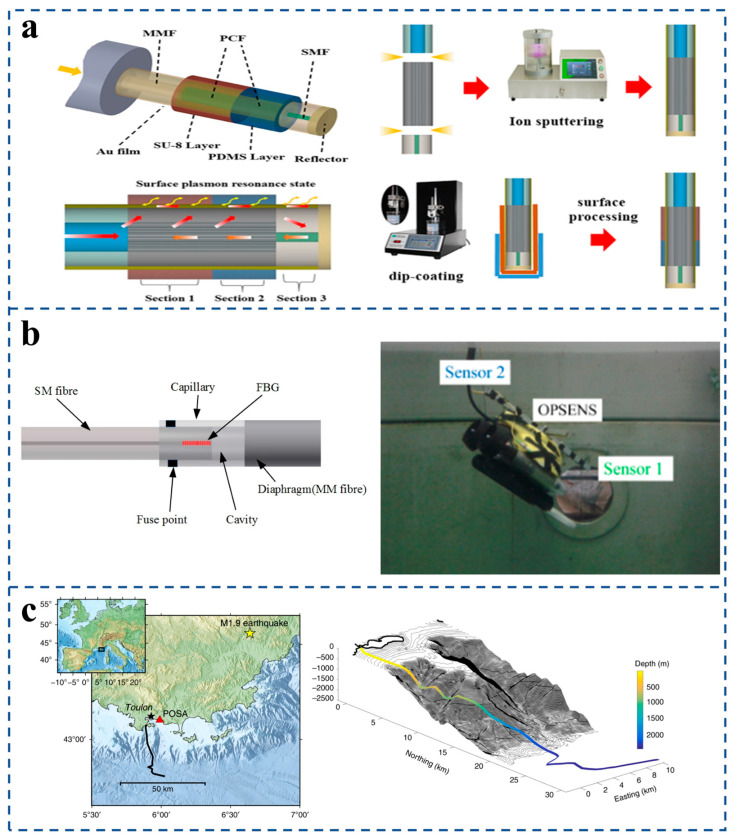
(**a**) An optical fiber sensor for the simultaneous measurement of salinity, temperature, and pressure in seawater (reprinted with permission from [118] © Elsevier, 2019). (**b**) Fiber-optic depth sensor for underwater vehicles (reprinted with permission from [111] © MDPI). (**c**) Distributed sensing system for earthquakes and ocean–solid (reprinted with permission from [112] © Springer Nature).

Zhao et al. [118] developed an optical fiber sensor for the simultaneous measurement of salinity, temperature, and pressure in seawater (Figure 13a). The sensor uses an integrated reflective optical fiber sensor to measure these three physical quantities. The Surface Plasmon Resonance (SPR) effect is generated by coating a gold film on the surface of the optical fiber, and the photonic crystal fiber (PCF) is used as an ideal mode excitation field. The slowly expanding cladding pattern provides multiple SPR resonance pits simultaneously. This design allows three distinct sensitive regions to be formed, and in the experiment, the maximum sensitivities for salinity, temperature, and pressure measurements were 0.560 nm/g/kg, 1.802 nm/°C, and 2.838 nm/MPa, respectively. This work provides a good example for the application of optical fiber structure in seawater salinity, temperature, and depth multi-parameter detection.

Dinesh et al. [111] designed fiber-optic depth sensors specifically for use in remotely operated underwater vehicles (Figure 13b). It is based on fiber extrinsic Fabry–Pérot interferometers combined with fiber Bragg gratings, EFPI providing pressure measurements, and FBG providing temperature measurements for temperature compensation. The sensor has excellent depth detection capability and can monitor depth changes of 0.02 m. This work has demonstrated that miniature OFPTS sensors can be used in marine environments. A. Sladen et al. [112] designed a distributed sensing system for earthquakes and ocean–solid earth interactions on submarine communication cables (Figure 13c). The method utilizes existing crisscross submarine optical cables as the carrier of seismic acoustic sensors, and Distributed Acoustic Sensors (DASs) measure 41.5 km of telecommunications cables. Observations show that, except for regional seismicity, the signal characteristics are comparable to those of coastal seismic stations. The advantage is that DASs only require access to one end of the submarine cable and provide dense spatial sampling of the wavefield, while the submarine cable has an extremely long service life, which allows the submarine image to be repeated over time to monitor changes in its characteristics for more effective earthquake and tsunami warnings. Fiber-optic pressure sensors build a deep blue sensing network covering the whole ocean by virtue of intrinsic corrosion resistance and passive sensing advantages. Its reliable data acquisition capability in extreme environments provides irreplaceable technical support for analyzing marine dynamic mechanism and early warning ecological risks and significantly deepens human scientific understanding of the complex system of the Earth.

### 5.4. Aerospace and Defense Field

In harsh conditions such as extreme temperatures, intense radiation, and ultra-high-speed dynamic loads, optical fiber pressure sensors have shown clear advantages. Their lightweight structure, natural resistance to electromagnetic interference, and fast millisecond-level response make them a key technology for intelligent sensing in aircraft and defense systems. Since they operate without a power supply, these sensors completely eliminate the risk of electromagnetic leakage from electronic systems. With embedded microstructures, they can monitor real-time pressure changes on the aircraft surface and analyze hypersonic airflow patterns. This capability offers reliable data for improving flight control, diagnosing structural health, and assessing stealth performance. As a result, the overall survivability and mission reliability of aerospace systems are greatly enhanced.

**Figure 14:**
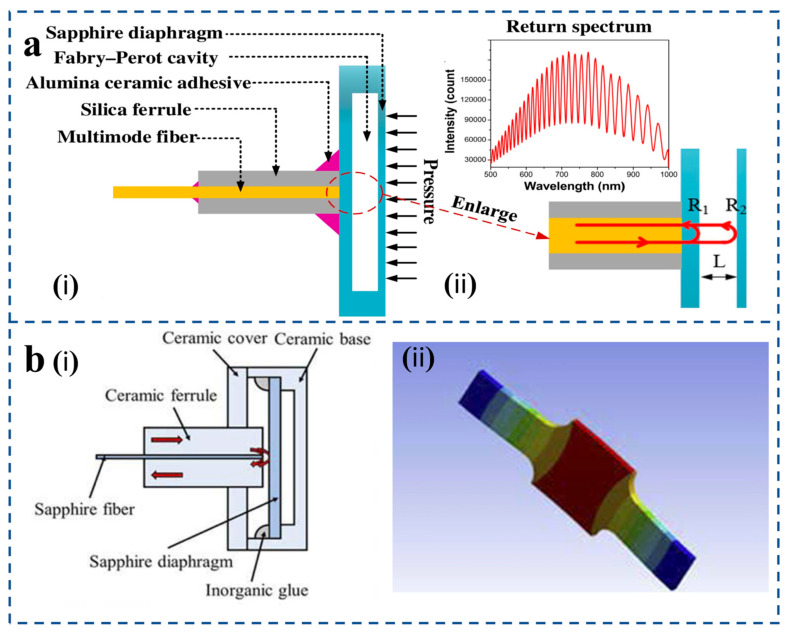
(**a**) Sensor configuration and principle of operation (reprinted with permission from [119] © Optical Society of America, 2019). (**i**) Schematic of the sapphire fiber-optic pressure sensor. (**ii**) FP cavity interference in the sensor. (**b**) (**i**) Sapphire optical fiber vibration sensor (reprinted with permission from [120] © Optics). (**ii**) Vibration mode simulation (reprinted with permission from [120] © Optics).

Li et al. [119] designed a high-temperature pressure sensor based on sapphire direct bonding (Figure 14a). The proposed sensor comprises a sensor head and a multimode fiber (core/cladding: 62.5/125 μm) [Figure 14a(i)]. The head is a two-layer sapphire structure with a thinned diaphragm and an etched cavity wafer, forming a sealed Fabry–Pérot (FP) cavity. The fiber is attached via a silica ferrule using high-temperature adhesive. Light reflects from the cavity base (R1) and diaphragm interior (R2), producing an interference spectrum used to determine the cavity length [Figure 14a(ii)]. The experimental results show that, like most other high-temperature pressure sensors, the sensor has temperature dependence, and the temperature coefficients of cavity length change and pressure sensitivity change are 1.25 nm/°C and 0.00025 nm/kPa ·°C, respectively.

Cui et al. [120] developed a high-temperature vibration sensor for use in extreme environments such as aerospace [Figure 14b(i)]. The sensor uses ceramic to fabricate a support structure and an etched single-sided polished sapphire wafer to be placed on top of the support. The FP cavity of the sensor consists of a sapphire fiber end face and a diaphragm polished face, at the same time, there is a unique sapphire diaphragm structure. The interference signal is picked up by a sapphire fiber. ANSYS simulations (Figure 14b(ii)) show that under perpendicular vibration, displacement increases from the supports toward the center of the diaphragm. The maximum and uniform displacement occurs in the central square region. The reflecting surface remains stable during vibration, significantly improving interference signal quality. The acceleration response of that sensor is linear along the range of 0–10 g, acceleration sensitivity is 20.91 nm/g, and the resonant frequency of the sensor is 2700 Hz. Because all the materials of the sensor are resistant to high temperature, it can work at 1500 °C, which provides a feasible method for vibration measurement in a high-temperature environment.

Optical fiber pressure sensor provides a new sensing mode for aerospace equipment. Its intrinsic reliability in extreme environments and synchronous resolution capability of multi-dimensional physical fields have become the key equipment for realizing the efficiency leap of advanced equipment such as the intelligent skin of hypersonic aircraft and long-term monitoring of spacecraft in orbit, injecting a new generation of sensing cornerstone into national defense security architecture.

### 5.5. Consumer Electronics and Smart Wearables Field

The optical fiber pressure sensor has gradually become a new research field of wearable devices through flexible optical waveguide structure and micro-scale integration technology. Its intrinsic biocompatibility eliminates the risk of skin sensitization caused by traditional metal sensors. Thanks to its high sensitivity and ability to analyze dynamic pressure distribution, this technology allows devices like smart watches to accurately track blood pressure trends. It also helps to create mechanical pressure maps for sports shoe soles and supports tactile feedback in VR systems. These features offer new, non-invasive ways to monitor health digitally and improve immersive human–computer interaction.

**Figure 15:**
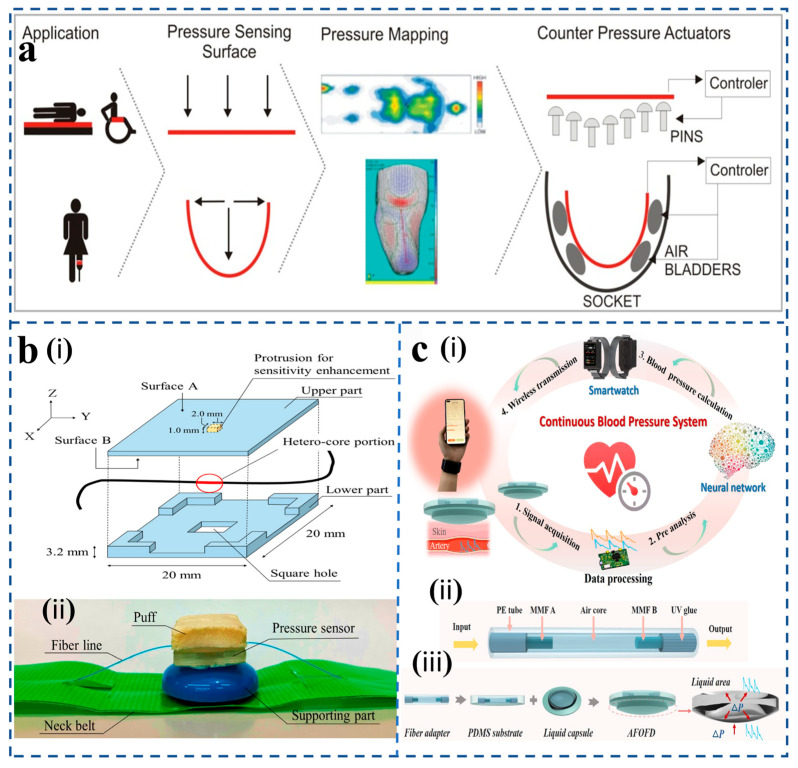
(**a**) Schematic of the pressure management system proposed for both cases of bed/seating system sensing surface (top row) and lower extremity prosthetic socket sensing surface (bottom row). The first step involves the measurement of the pressure loads across the sensing surface, then generating pressure maps that define pressure distribution, and finally driving counter-pressure actuators to redistribute pressure across the surface for pressure relief (reprinted with permission from [121] © IntechOpen). (**b**) A fiber-optic non-invasive swallowing assessment device based on a wearable pressure sensor (reprinted with permission from [122] © MDPI). (**i**) Structure of the pressure sensor using hetero-core fiber optics. (**ii**) Wearables wallowing test device. (**c**) Schematic of the blood pressure monitoring system (reprinted with permission from [113] © Springer Nature). (**i**) The monitoring system consists of an optical fiber sensor, signal acquisition, data processing, blood pressure calculation, smartwatch terminal, and mobile phone. (**ii**) The fiber adapter’s construction. (**iii**) The fabrication of the proposed sensor.

Pissadakis et al. [121] introduces a flexible optical fiber-based pressure-sensing surface utilizing fiber Bragg gratings (FBGs) embedded in a PDMS polymer sheet, designed for real-time pressure monitoring at critical human–machine interfaces such as prosthetic sockets, medical beds, and wheelchairs to prevent pressure ulcers; the system operates by measuring interface pressure loads, generating detailed distribution maps, and dynamically activating counter-pressure actuators to redistribute excessive loads, all while offering high sensitivity, minimal hysteresis, and a compact, portable design suitable for demanding biomedical applications (Figure 15a).

Maeda et al. [122] introduces a wearable swallowing assessment device based on a hetero-core fiber-optic pressure sensor, which non-invasively detects laryngeal movement during swallowing. The sensor consists of a 9 µm core single-mode fiber with a 1.7 mm-long, 5 µm core hetero-core segment fusion-spliced in the middle, embedded in a silicone rubber housing featuring a 2 mm^2^ protrusion for enhanced sensitivity and a square hole in the base to facilitate bending [Figure 15b(i)]. This structure converts external pressure into curvature changes of the hetero-core region, resulting in measurable optical loss. The sensor demonstrated high sensitivity (0.592 dB/kPa) and excellent linearity (R^2^ = 0.995).

The wearable device, which includes a soft puff for comfort and a neck belt for secure placement, successfully captured characteristic swallowing waveforms with two valleys in subjects across different age groups, enabling the detection of age-related changes in swallowing function [Figure 15b(ii)]. Li et al. [113] presents an optical-fiber-sensor-assisted smartwatch for automatic and continuous blood pressure monitoring, addressing limitations of existing wearables like position sensitivity and electromagnetic interference. The system [Figure 15c(i)] integrates a fiber-optic sensor for pulse wave detection, signal processing, and wireless data transmission to a smartphone app. The sensor core [Figure 15c(ii)] is a fiber adapter comprising two multimode fibers with different core diameters inserted into a polyethylene (PE) tube, forming an air cavity whose deformation under pressure modulates transmitted light intensity. The fabrication process [Figure 15c(iii)] involves assembling and sealing the fibers with UV glue, then sandwiching the adapter between flexible PDMS films and integrating it into a liquid-filled capsule that enhances spatial insensitivity and body conformality via Pascal’s Principle. The smartwatch demonstrates high sensitivity (−213 µW/kPa), fast response (5 ms), excellent durability (70,000 cycles), and clinical-grade accuracy (SBP: −0.35 ± 4.68 mmHg, DBP: −2.54 ± 4.07 mmHg) by combining pulse wave feature extraction with a machine-learning model, enabling reliable 24/7 ambulatory blood pressure monitoring.

Fiber-optic pressure sensors are leading the shift from traditional consumer electronics sensing toward multimodal fusion and interference-free operation. Their potential for micro- and nanoscale integration, along with the ability to harvest environmental energy, opens up new opportunities for how humans interact with machines. This technology could create a fresh sensing framework to support personalized digital health twins, immersive experiences in the metaverse, and smart, connected textiles.

## 6. Current Challenges and Trends

### 6.1. Current Challenges

The development of fiber-optic pressure sensors still faces the following problems to be solved, such as the following.

Fiber-optic pressure sensors face serious challenges when exposed to extreme environments like high temperatures above 300 °C and strong radiation. At such high heat, the usual polymer coating on the fiber can break down and carbonize, which weakens the fiber’s mechanical strength. Although the fiber’s core is made of quartz with a high melting point, it still experiences changes in refractive index at continuous high temperatures. This leads to errors in wavelength measurements. When the temperature goes beyond 500 °C, quartz glass suffers from a photon darkening effect, causing significant light loss. Even more concerning is the combined effect of repeated temperature and pressure changes. This accelerates the growth of tiny cracks in the fiber and can eventually cause the sensor to fail. These problems limit how reliably fiber-optic sensors can be used in important areas like aero-engines.

Another major challenge for optical fiber pressure sensors is their cross-sensitivity to multiple physical parameters, making it difficult to isolate pressure signals from other influences. For example, temperature changes can shift the central wavelength of fiber Bragg gratings (FBGs) by several times more than the shift caused by pressure alone. This happens due to both the thermo-optic effect and thermal expansion. In fluid environments, changes in the refractive index of the medium affect the evanescent field coupling, causing signal drift that can be mistaken for pressure variation. For microbend sensors, external vibrations create dynamic strain, which distorts the pressure spectrum through the chirp effect. Additionally, transverse stress can introduce birefringence in the fiber, leading to over 20% error in polarization-sensitive pressure readings. These interactions between multiple physical fields create complex nonlinear behavior. Even with sensor arrays and compensation algorithms, achieving stable decoupling with sub-kPa accuracy remains a significant technical barrier.

Current optical fiber pressure sensors face limits in dynamic response and bandwidth. When capturing fast-changing pressure signals, they often show response lag. For example, microbend structures that rely on elastic diaphragms are affected by material creep, leading to a 10–15% drop in signal amplitude under millisecond-level pressure changes. Fabry–Pérot interferometric sensors also face constraints. Because of limitations in how the cavity length can be adjusted, their resonant frequency typically stays below 50 kHz. This is not fast enough for analyzing hypersonic flow fields. A deeper issue lies in the polymer encapsulation layer. When pressure frequencies go beyond 10 kHz, the viscoelastic nature of the protective material causes an acoustic mismatch. This mismatch significantly reduces how much stress actually reaches the fiber core, weakening the sensor’s ability to detect high-speed pressure changes accurately.

Miniaturization remains a major bottleneck for fiber-optic sensors, especially in fields like consumer electronics and implantable medical devices. Although traditional optical fibers have a diameter of 125 μm, this size is still too large for applications such as vascular pressure monitoring. Reducing the diameter below 80 μm helps with flexibility but causes the fiber’s mechanical strength to drop by two-thirds, making it more prone to microbending losses in wearable settings. For multi-parameter sensing, multiple gratings or interference structures need to fit within a very small volume—often less than a cubic millimeter. However, limited micromachining precision leads to high signal crosstalk, sometimes reaching 25%, which affects accuracy. A more serious issue is the fragile nature of optical fibers. They are not well suited for integration with standard electronic packaging techniques. As a result, achieving wafer-level integration with CMOS technology remains difficult. This limits the potential for low-power, compact systems required for fully wireless wearable devices.

High-precision optical fiber pressure sensors are still costly, mainly due to the use of special materials and the need for precision manufacturing. For example, radiation-hardened optical fibers cost over 50-times more than standard ones. Equipment for writing gratings with femtosecond lasers can cost more than USD 1 million. Although microstructured fibers like photonic crystal fibers offer much better pressure sensitivity, the production yield of their complex preforms is below 30%. In the system side, demodulation requires a narrow-linewidth laser with a wavelength stability better than 1 pm, along with a spectrometer capable of 0.1 pm resolution. This causes the cost of a single sensing channel to exceed USD 10,000. These technical and economic barriers make it difficult to deploy such sensors on a large scale.

### 6.2. Future Development Trends

The future development of fiber-optic pressure sensors will rely heavily on integration with smart materials. Designs based on metamaterial photonic crystal fibers can overcome the optical limitations of traditional quartz. By introducing engineered microstructures, these sensors can achieve a 10 to 100-times improvement in pressure sensitivity while naturally reducing temperature-related signal interference. Some of the most advanced research is now exploring self-healing polymer optical waveguides. These materials can trigger repair processes automatically when microcracks begin to form, potentially extending the service life beyond ten years. These material innovations will enable fiber sensors to operate reliably in extreme environments.

The next generation of optical fiber sensors will move beyond single-parameter pressure monitoring toward collaborative sensing across multiple physical fields. With their ultra-wide spectral range, fiber sensors can detect pressure-induced strain, temperature changes, acoustic vibrations, and variations in the refractive index of surrounding media, all at the same time. By combining these signals with deep learning algorithms, it becomes possible to decouple multiple parameters in real time directly within the optical domain. For instance, in smart oil fields, distributed fiber networks can monitor wellbore pressure changes and formation microseismic activity. Coupled with fluid simulation models, this data can help to track reservoir dynamics and improve oil recovery. In smart city infrastructure, buried fiber sensors can capture data on traffic loads, soil stress, and pipeline pressure.

Traditional fiber gratings are made point by point with UV lasers, which is slow and depends on the fiber’s photosensitivity. Femtosecond laser writing solves this by creating 3D structures in non-sensitive fibers, speeding up the process over tenfold and even working on heat-resistant materials like sapphire. Techniques like electron beam lithography and ion beam milling can carve tiny air hole patterns into photonic crystal fibers. This helps to boost pressure sensitivity while keeping temperature effects low. Meanwhile, roll-to-roll nanoimprinting could allow large-scale production of flexible fiber sensing films, cutting costs for smart fabrics by up to 90%. Together, these new methods are turning fiber-optic pressure sensors from lab tools into scalable, industrial products—ready for big markets like consumer electronics and smart healthcare.

## 7. Conclusions

This paper looks at recent progress in fiber-optic pressure sensors, especially how new microstructures and better manufacturing methods are making them more effective. Designs using things like micro-cavities and gratings have boosted their sensitivity, speed, and range. Because they are safe, small, and work well in tough environments, they are a good fit for situations with high heat, pressure, or corrosion. New materials and micro-scale processing are also helping to improve their performance and make them more adaptable. Overall, fiber-optic pressure sensors are becoming more important in sensing technology. With continued improvements, they will likely play a bigger part in areas like healthcare, smart devices, and safety systems.

## Figures and Tables

**Table 1:** Performance Indicators and Importance of Optical Fiber Pressure Sensors.

Parameter	Typical Range	Significance
Pressure Range	0–100 MPa	Determines the application domain (biomedical, industrial, aerospace)
Sensitivity	1–50 pm/kPa	Affects the accuracy in detecting small pressure changes
Resolution	0.01–1 kPa	Critical for detecting subtle variations in pressure
Response Time	1–100 ms	Important for real-time monitoring
Dynamic Range	40–80 dB	Needed for broad pressure variations
Operating Temperature	−40–2500 °C	For aerospace and harsh environments

## Data Availability

No new data were created.
